# Transcriptional response of rice flag leaves to restricted external phosphorus supply during grain filling in rice cv. IR64

**DOI:** 10.1371/journal.pone.0203654

**Published:** 2018-09-13

**Authors:** Kwanho Jeong, Omar Pantoja, Abdul Baten, Daniel Waters, Tobias Kretzschmar, Matthias Wissuwa, Cecile C. Julia, Sigrid Heuer, Terry J. Rose

**Affiliations:** 1 Southern Cross Plant Science, Southern Cross University, Australia; 2 Southern Cross GeoScience, Southern Cross University, Australia; 3 Instituto de Biotecnología, Universidad Nacional Autónoma de México, Cuernavaca, Morelos, Mexico; 4 ARC ITTC for Functional Grains, Charles Sturt University, Wagga Wagga NSW, Australia; 5 Genotyping Services Laboratory, International Rice Research Institute (IRRI), Metro Manila, Philippines; 6 Crop, Livestock and Environment Division, Japan International Research Center for Agricultural Sciences, Ohwashi, Tsukuba, Ibaraki, Japan; 7 Department of Plant Biology and Crop Sciences, Rothamsted Research, West Common, Harpenden, Herts, United Kingdom; Louisiana State University College of Agriculture, UNITED STATES

## Abstract

Plant phosphorus (P) remobilisation during leaf senescence has fundamental implications for global P cycle fluxes. Hypothesising that genes involved in remobilisation of P from leaves during grain filling would show altered expression in response to P deprivation, we investigated gene expression in rice flag leaves at 8 days after anthesis (DAA) and 16 DAA in plants that received a continuous supply of P in the nutrient solution vs plants where P was omitted from the nutrient solution for 8 consecutive days prior to measurement. The transcriptional response to growth in the absence of P differed between the early stage (8 DAA) and the later stage (16 DAA) of grain filling. At 8 DAA, rice plants maintained production of energy substrates through upregulation of genes involved in photosynthesis. In contrast, at 16 DAA carbon substrates were produced by degradation of structural polysaccharides and over 50% of highly upregulated genes in P-deprived plants were associated with protein degradation and nitrogen/amino acid transport, suggesting withdrawal of P from the nutrient solution led to accelerated senescence. Genes involved in liberating inorganic P from the organic P compounds and vacuolar P transporters displayed differential expression depending on the stage of grain filling stage and timing of P withdrawal.

## Introduction

An estimated 5.7 billion ha of global crop-lands are deficient in bioavailable phosphorus (P) [1]. To obtain high yield and maintain soil fertility, regular inputs of P fertiliser, and other nutrients, are required. The efficiency with which applied P fertiliser is utilised by crops is, however, generally low. Much of the applied P fertiliser becomes ‘fixed’ with iron (Fe) and aluminium (Al) oxides and hydroxides in acid soils, and as calcium (Ca) complexes in alkaline soils [2], which is problematic given that rock phosphate utilised by most P-fertilisers is a finite natural resource [3].

A further factor that contributes to unsustainable P use in agriculture is the removal of an estimated 12 million tonnes of P in harvested agricultural produce each year [4, 5], of which little is recycled back to fields [3]. Most of the P removed in harvested produce is in the grains of cereal crops [5], mostly in the form of the anti-nutrient phytate which cannot be digested by humans and other monogastric animals [6]. Reducing P levels in the grains of cereals would minimise the amount of P removed from fields at harvest and, if crop stubble is retained in the field, the P fertiliser requirements of subsequent crops would be reduced in the long term [7, 8].

Recent studies have identified critical periods of P remobilisation from leaves and loading into developing grains of rice (*Oryza sativa* L.) [9], however, our understanding of the molecular regulation of this process is limited [10–13]. Using rice as a model, we recently investigated whether a subset of genes involved in the well described molecular response of plants to P starvation [14–16] were involved in the remobilisation of P from senescing flag leaves to developing rice grains [10]. Three purple acid phosphatases (*OsPAP3*, *OsPAP9b* and *OsPAP10a*) that have been implicated in the P starvation response were significantly upregulated during the phase of rapid P remobilisation from flag leaves (15 days after anthesis; DAA) compared to 6 DAA when flag leaf P levels were relatively stable [10]. Additionally, three genes not previously associated with the P starvation response, *OsPAP26*, *SPX-MFS1* and *SPX-MFS2*, showed expression profiles consistent with an involvement in P remobilisation from senescing flag leaves [10]. Consistent with these findings, the *Arabidopsis* homologue of *OsPAP26*, *AtPAP26*, was shown to be involved in P remobilisation from senescing *Arabidopsis* leaves [17]. In addition, data from recent studies suggest that SPX genes may encode vacuolar P transporters (VPTs) [18] and two VPTs were recently identified in rice as influx (*OsSPX-MFS1*) and efflux (*OsSPX-MFS3*) transporters [19–21].

While the expression profiles of the *Os*PAPs and *Os*SPX-family genes were consistent with a role in remobilisation of P from senescing rice flag leaves [10], the absence of any specific P treatment prevented us from establishing a definitive link with P remobilisation. In a subsequent study, we found that P from specific leaf P pools was remobilised prematurely following the permanent withdrawal of P from the nutrient solution at anthesis or 8 DAA [22]. The data suggested that the demand for P in developing grains led to premature remobilisation of P from flag leaves in the absence of an external (nutrient solution) P supply.

The aim of the present study was to investigate whether the premature remobilisation of P in flag leaves when external P supply was withdrawn during grain filling corresponded to changes in expression of:

1. previously identified genes putatively related to P remobilisation during senescence including *Os*PAPs and *Os*SPX genes from the study of Jeong *et al*.[10] and;
2. novel genes that have not previously been associated with P remobilisation from senescing leaves

This was tested using an RNA-seq approach to compare gene expression in flag leaves of rice at specific time points during grain filling from plants that had a continuous P supply until maturity and from plants that had P permanently withdrawn (for 8 d) from the hydroponic solution at anthesis and at 8 DAA, respectively. We used the rice mega-variety IR64 that has been well-characterised genetically and is cultivated on more than 10 million ha across the globe [23].

## Materials and methods

### Experimental design and overview

Rice plants (cv. IR64) were grown to maturity in hydropic culture solution under adequate P supply (0.75 mg P day^-1^), supplied until maturity, or P permanently withdrawn from the nutrient solution at anthesis or 8 DAA. The amount of P provided in the nutrient solution for ‘adequate’ P supply was determined in a preliminary experiment, and was defined as the minimum supply of P required for obtaining maximum grain yields. Data from the preliminary experiment, along with the flag leaf photosynthesis and P mobilisation data, are presented in Jeong *et al*. [22], and key data pertinent to this paper are presented in S1 Table.

### Plant growth and experimental design

Evenly sized rice seeds (cv. IR64) were sterilised with HClO_3_ for 2 min and germinated in Petri dishes in the dark at 30°C for 2 d. The germinated rice seeds were transferred to a mesh floating on a hydroponic nutrient solution containing 1 mM calcium (CaCl_2_) and 36 μM iron (Fe EDTA). After 10 days, the solution was changed to half strength Yoshida solution [24] without P, in which the plants were grown for a further 2 weeks. Nutrient solutions were replaced after 1 week. After 2 weeks growing on the floating mesh, two evenly sized seedlings were transplanted into 5 L pots wrapped with aluminium foil containing full strength Yoshida solution without P. Yoshida nutrient solution was changed and the pH adjusted to 5.2–5.5 each week. Rice plants were grown under temperature-controlled conditions in a glasshouse at Southern Cross University (Lismore, NSW, Australia) with a mean day/night air temperature of 29°C/21°C and relative humidity (RH) of 75%.

Plants received 0.75 mg of P per day per pot by application of 17.5 ml of P stock solution (150 mg P L^-1^) every 3.5 days to the nutrient solution. Phosphorus withdrawal treatments were applied during the reproductive growth phase as shown in S1 Fig. Each treatment was replicated three times. Anthesis was defined as the state when 50% of panicles had at least 50% of florets with visible anthers. A set of six panicles that reached the booting stage simultaneously were tagged and their flag leaves were harvested for RNA extraction and RNA-seq analysis.

### Total RNA extraction, library construction and sequencing

Based on flag leaf photosynthesis and P mobilisation data (S1 Table) [22], two sets of samples that had received ‘adequate’ P supply were selected for RNA-seq analysis. The first set consisted of flag leaf samples harvested at 8 DAA from plants where P supply had been withdrawn at anthesis (T8) and of the corresponding control plants (P supplied continuously; C8). The second set consisted of leaf samples from plants harvested at 16 DAA where P supply had been withdrawn at 8 DAA (T16) and plants of the corresponding control treatment (P supplied continuously; C16) (S1 Fig). These two time-points were chosen because in response to P deprivation, photosynthesis was unaffected in T8 while it was significantly impaired in T16 (S1 Table) [22].

Total RNA was extracted using the RNeasy Mini kit (Qiagen, Victoria, Australia) according to the manufacturer’s instructions. After extraction, total RNA was quantified with a Nanodrop (ND1000, Labtech, Paris, France) and the quality of RNA was examined on a 2100 Bioanalyzer (Agilent Technologies, California, US). Based on the quantity and quality control of RNA samples, high quality RNA samples were selected for library construction. Samples that did not meet the criteria (i.e. 2.0 ≥ 260 / 280 ratio, 7.0 RIN number) were re-extracted. Library construction using Truseq RNA V2 kit (Illumina, California, US) and RNA sequencing (RNA-seq) using Hi-seq 2500 system (Illumina) were carried out by Macrogen (Seoul, Korea).

### Analysis of RNA-seq data

RNA-seq data analysis was performed as described in Jeong *et al*. [10]. Fastq files were filtered for adapter sequence, poly-N stretches and low-quality reads using FASTQC [25] and the BBDuk module of the BBMap software package (https://sourceforge.net/projects/bbmap/, version 35.51). Bowtie version 2.2.4 [26] was used to index the rice genome (IRGSP 1.0). Clean high quality reads were mapped using the splice aware RNASeq aligner program TopHat version 2.1.0 [27]. The Ensembl Plants (http://plants.ensembl.org/Oryza_sativa/Info/Annotation) *O*. *sativa* cv. Nipponbare (ssp. *japonica*) reference genome annotation was utilised. TopHat identified the exon–exon junctions and produced the read vs genome alignment in BAM (Binary Alignment Map) format. Cufflinks [28] then used the TopHat-generated alignment to assemble a set of reference-based transcripts. Finally, the CuffDiff module of Cufflinks was used to identify differentially expressed genes between samples. Raw sequencing data have been uploaded to SRA (Sequence Read Archive) database (accession number: SRP134062).

A total of 304 million 101 bp paired end reads generated more than 96% of high quality reads across all libraries and about 87.6% of those high quality reads were mapped to the reference rice genome (S2 Table).

Gene expression levels were normalised using the FPKM (Fragments Per Kilobase of transcript per Million mapped reads) method. Differentially expressed genes (DEGs) were calculated by comparing the FPKM values between T8 vs C8 and T16 vs C16 using three biological replicates for each treatment. Significant DEGs were defined as those genes that were up or downregulated with log_2_ fold change > 1 or < -1, respectively, while significance threshold was set at a false discovery rate (FDR) corrected *p* value of < 0.05.

### Gene identification, annotation and classification

A total of 400 DEGs, the 100 most differentially up- and down-regulated genes from both T8 and T16, were categorised based on their descriptions, annotated functions and data from the literature (S3 and S4 Tables). After sorting based on log_2_ fold change the annotations were retrieved from the rice annotation project database (RAP-DB; http://rapdb.dna.affrc.go.jp/index.html), rice locus identifier search (http://rice.plantbiology.msu.edu/analyses_search_locus.shtml) and RiceXpro global gene expression profile (http://ricexpro.dna.affrc.go.jp/category-select.php). The functional annotation of DEG products was cross referenced with the functional protein association network String (version 10.0; http://string-db.org/). To annotate and classify transcription factors (TFs), plant transcription factor database version 3.0 (PlantTFDB; http://planttfdb.cbi.pku.edu.cn/) was used.

### Examination of key genes in the P starvation response

We assumed that the withdrawal of P from the nutrient solution during grain filling would not simply elicit the well-known P starvation response observed when young plants are deprived of P. To confirm this, we specifically examined the expression of 11 key genes (*OsPHO1*, *OsPHO2*, *OsPHR1*, *OsPHR2*, *OsSPX2*, *OsSPX3*, *OsSPX5*, *OsPT5*, *OsPAP23*, *OsIPS1* and *OsACP1*) that have been widely reported to respond to P starvation in young rice plants (Table 1) [16, 29–32].

**Table 1:** Gene expression profile of eleven key P starvation response (PSR) genes in rice flag leaves at 8 or 16 days after anthesis, following P withdrawal from the nutrient solution 8 d earlier.

Gene ID (MSU)	Gene name	8DAA (log_2_)		16DAA (log_2_)	
		Up	Down	UP	Down
LOC_Os05g48390	*OsPHO2*	0.52	-	0.57	-
LOC_Os01g52230	*OsACP1*	-	-0.73	-	-1.69
LOC_Os03g05334	*OsIPS1*	1.34	-	-	-1.5
LOC_Os08g17784	*OsPAP23*	1.27	-	-	-
LOC_Os04g10690	*OsPT5*	-	-0.79	-	-
LOC_Os02g10780	*OsSPX2*	-	-	-	-
LOC_Os10g25310	*OsSPX3*	-	-	-	-
LOC_Os03g29250	*OsSPX5*	2.13	-	-	0.76
LOC_Os03g21240	*OsPHR1*	-	-	-	-
LOC_Os01g02000	*OsPHO1*	-	-1.53	-	-
LOC_Os07g25710	*OsPHR2*	-	-0.58	-	-

### Expression of specific genes of interest from earlier studies

We previously observed that 32 genes, including P starvation response genes (PSRs), OsPAPs, rice P transporters (OsPTs), and OsSPX and OsSPX-MFS family genes, were upregulated in rice flag leaves at 15 DAA when leaves were acting as a P source [10]. We further investigated the expression of these 32 genes (hereafter referred to as the ‘32 P-remobilisation related genes (PRGs)’; see list in S5 Table) in the present study at 8 DAA and 16 DAA in both treatment (P withdrawn) and control samples. Further, given the critical role of Pi transporter family 1 (PHT1) genes in Pi uptake from roots and remobilisation in vegetative tissues [33], the expression of PHT1 genes (*OsPT1* to *OsPT13*) was analysed at 8 DAA and 16 DAA.

## Results

### Global gene expression data and differential gene expression

**Fig 1:**
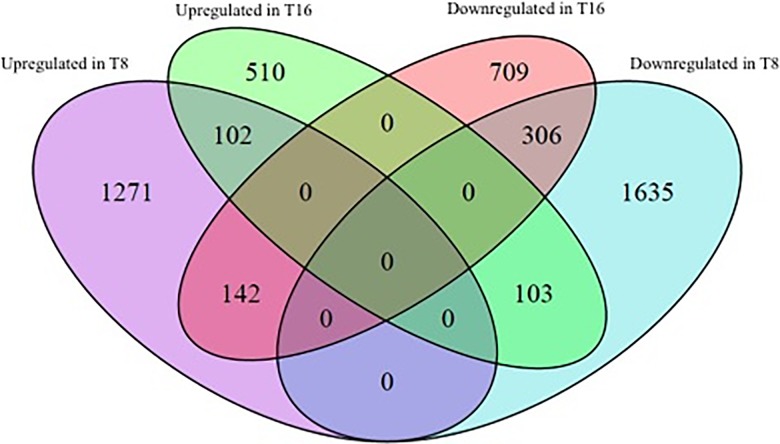
Venn diagram of total DEGs and overlapping DEGs (log_2_ fold change > +1 or < -1, *P* value < 0.05).

Three biological replicates for each library (T8, C8, T16 and C16) were analysed using RNA-seq. The average number of high quality reads of three replicates from each library, T8, C8, T16 and C16, were 24 ± 0.35, 25 ± 4.84, 26 ± 1.37 and 23 ± 0.97 million, respectively. An average of 87.6 ± 0.69% of high quality reads from each replicate were mapped to the rice genome (S2 Table) and distinct DEGs retrieved based on log_2_ fold change (log_2_ > 1 or < -1). At 8 DAA, 1,515 and 2,044 genes were up and downregulated, respectively, while 715 and 1,157 genes were up and downregulated, respectively, at 16 DAA (Fig 1). Subsequently, the DEGs common to both treatments were identified, with 102 DEGs upregulated at both 8 and 16 DAA, while 306 DEGs were downregulated at both 8 and 16 DAA (Fig 1). The number of DEGs that were upregulated at 8 DAA but downregulated at 16 DAA was 142, and 103 DEGs were upregulated at 16 DAA but downregulated at 8 DAA (Fig 1).

### Expression of key P starvation induced genes

We assumed that P withdrawal would not simply elicit the up-regulated expression of PSR genes. In order to verify this, the expression of 11 highly and consistently expressed PSR genes was investigated. Seven of the 11 key PSR genes examined, including the transcription factors *OsPHR1* and *OsPHR2*, were either not differentially expressed or were downregulated in T8 and T16 (Table 1). Only *OsPHO2* was upregulated in both T8 and T16, with log_2_ fold changes of 0.52 and 0.57, respectively, while *OsIPS1*, *OsPAP23* and *OsSPX5* were upregulated in T8 (Table 1).

### Expression of 32 PRGs putatively involved in P remobilisation during grain filling

In an earlier study [10], we found that the expression profiles of 32 P-related genes (PRGs) were consistent with a possible role in remobilisation of P from senescing rice flag leaves, however, the absence of any specific P treatment precluded any definitive conclusions. Three of the 32 PRGs (*OsPAP15*, *OsPAP10a* and *OsPAP1d*) were significantly upregulated in T8 while five genes (*OsSPX-MFS1*, *OsSPX-MFS3*, *OsPHO1;1*, *OsPAP9b* and *OsCAX1a*) were downregulated (Table 2). In T16, only one gene (*OsSPX-MFS3*) was upregulated while three genes (*OsPT20*, *OsPT19* and LOC_Os03g15530) were downregulated (Table 2). Most of the 32 PRGs were not differentially expressed; only *OsSPX-MFS1* was highly downregulated in T8 with a log_2_ fold change of -3.13 (Table 2). *OsSPX-MFS2* and *OsSPX-MFS4* were not differentially expressed in flag leaves during grain filling under P withdrawal (S2 Fig).

**Table 2:** Differential gene expression among the 32 PRGs^ǂ^ in rice flag leaves at 8 or 16 days after anthesis, following P withdrawal from the nutrient solution 8 d earlier.

Time point	Gene ID (MSU)	Gene name	FPKM		log_2_ *	P value
			Treatment (-P)	Control (+P)		
	LOC_Os03g63074	*OsPAP15*	12.19	4.87	1.32	5.00E-05
	LOC_Os01g56880	*OsPAP10a*	61.58	25.63	1.26	5.00E-05
	LOC_Os12g38750	*OsPAP1d*	33.39	14.84	1.17	5.00E-05
T8	LOC_Os04g48390	*OsSPX-MFS1*	0.54	4.77	-3.13	5.00E-05
	LOC_Os06g03860	*OsSPX-MFS3*	8.08	27.75	-1.78	5.00E-05
	LOC_Os01g02000	*OsPHO1;1*	0.38	1.10	-1.53	5.00E-05
	LOC_Os01g58640	*OsPAP9b*	5.46	14.99	-1.46	5.00E-05
	LOC_Os01g37690	*OsCAX1a*	33.29	75.63	-1.18	5.00E-05
	LOC_Os06g03860	*OsSPX-MFS3*	31.63	11.84	1.42	5.00E-05
T16	LOC_Os09g38100	*OsPT20*	0.72	2.03	-1.51	5.00E-05
	LOC_Os09g28160	*OsPT19*	0.81	1.92	-1.24	5.00E-05
	LOC_Os03g15530	Expressed gene	2.13	4.39	-1.04	5.00E-05

ǂ Gene expression in 20 genes out of 32 PRGs did not change upon P withdrawal

* Positive and negative values indicate upregulation and downregulation, respectively.

### Analysis of the 100 most highly up-regulated and down-regulated DEGs in T8 and T16

In order to obtain information on the metabolic pathways or processes that were responding to P-withdrawal, we decided to analyse the 100 most highly up/downregulated genes in T8 and T16 on flag leaves under P withdrawn between different time-points during rice grain filling.

#### Highly upregulated genes at 8 DAA in response to P-withdrawal

**Fig 2:**
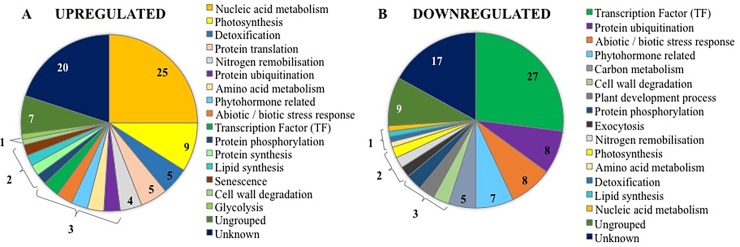
**The 100 most highly upregulated (A) and downregulated (B) genes in T8.** The number in the chart indicates the number of genes that are assigned in the category.

More than 50% of the upregulated genes in T8 were classified within the nucleic acid metabolism, photosynthesis, detoxification and protein translation groups (Fig 2A). Twenty-five of the genes upregulated in T8 were assigned to nucleic acid metabolism (Fig 2A). Most of these were broadly associated with DNA replication and RNA splicing in the chloroplast, for example, ribonucleotide reductase (RNRL1; LOC_Os06g07210) and chloroplastic group IIA intron splicing facilitator CRS1 (LOC_Os05g47850). Additionally, six genes encoding for proteins containing pentaricopeptide repeat (PPR) domains, four encoding for proteins with a glycine rich (GRP) domain involved in RNA recognition and RNA polymerases, that together are involved in RNA transcription and editing in chloroplast, were also upregulated in T8 (S3 Table). Nine DEGs were assigned to photosynthesis (Fig 2A). Among these were photosystem II (PSII) oxygen evolving complex protein PsbQ family protein (LOC_Os02g36850, with a log_2_ fold change of 2.6). The light-harvesting chlorophyll a/b-binding proteins from photosystem I (PSI) precursor (*Lhca6*, LOC_Os09g26810), ferredoxin-type domain containing protein (LOC_Os07g30670) and aldose 1-epimerase family protein (AEP; LOC_Os03g53710), with log_2_ fold changes of 3.0, 4.0 and 3.2, respectively (S3 Table).

Genes involved in detoxification processes were also upregulated in T8 (Fig 2A), including thioredoxin and oxidoreductase proteins that are involved in detoxification of reactive oxygen species (ROS) in chloroplasts (S3 Table). In addition, four genes associated with nitrogen (N) remobilisation were upregulated, including the urea transporter, *OsTIP4;1* (LOC_Os05g14240) and aspartate carbamoyltransferase (LOC_Os08g15030), that catalyses the first committed step in pyrimidine biosynthesis, the condensation of l-aspartate and carbamoyl phosphate to form N-carbamyl-L-aspartate and inorganic phosphate (S3 Table).

Five ribosomal and translational proteins were upregulated and were assigned to protein translation and several other upregulated DGEs were assigned to protein related processes such as protein synthesis, phosphorylation and ubiquitination (Fig 2A). Descriptions of other genes that were upregulated in T8 are given in S3 Table.

#### Highly down regulated genes at 8 DAA in response to P-withdrawal

Twenty-seven of the genes that were downregulated in T8 were TFs, five of which participate in protein synthesis regulation. For example, *OsbHLH148* (LOC_Os03g53020) acts on an initial response of jasmonate-regulated gene expression toward drought tolerance; *OsDof2* (LOC_Os01g15900) is associated with plant growth, leaf expansion, grain size and flowering time in rice. The heat stress transcription factor, *OsHsfC2a* (LOC_Os02g13800) was downregulated in T8. The WRKY transcription factors *OsWRKY21* (LOC_Os01g60640) that putatively regulates a transcriptional repressor of the gibberellin signalling pathways, and *OsWRKY71*, associated with the defensive reaction to rice blast disease were also downregulated in T8 (Fig 2B and Table 3).

**Table 3:** The expression profile of downregulated transcription factor families in the 100 most differentially expressed genes in T8.

Gene ID		Gene name		Description		FPKM				Log_2_	
						Treatment		Control			
**Jasmonate signalling**											
LOC_Os04g23550		*OsbHLH006*		Helix-loop-helix DNA-binding domain containing protein		0.32		23.15		-6.18	
LOC_Os10g25230		*OsJAZ13*		Tify domain containing protein, ZIM domain containing protein		0.53		23.29		-5.45	
LOC_Os03g08330		*OsJAZ10*		Tify domain containing protein, ZIM domain containing protein		6.2		155.07		-4.64	
LOC_Os10g25290		*OsJAZ1*		Tify domain containing protein, ZIM domain containing protein		1.75		27.40		-3.97	
LOC_Os03g08310		*OsJAZ9*		Tify domain containing protein, ZIM domain containing protein		0.29		4.52		-3.96	
**Ethylene response**											
LOC_Os08g36920		*OsERF104*		AP2 / ERF domain containing protein, expressed		0.85		41.45		-5.6	
LOC_Os02g45450		*OsERF25*		Dehydration-responsive element-binding protein, putative, expressed		1.52		43.45		-4.84	
LOC_Os02g45420		*OsERF20*		AP2 / ERF domain containing protein, expressed		0.05		0.91		-4.2	
LOC_Os10g41330		*OsERF96*		AP2 / ERF domain containing protein, expressed		9.84		140.52		-3.84	
LOC_Os09g35010		*OsERF31*		Dehydration-responsive element-binding protein, putative, expressed		20.33		280.25		-3.78	
LOC_Os09g35030		*OsERF24*		Dehydration-responsive element-binding protein, putative, expressed		4.69		63.76		-3.77	
LOC_Os03g09170		*OsERF47*		AP2 / ERF domain containing protein, expressed		0.92		11.89		-3.69	
LOC_Os01g66270		*OsERF17*		AP2 / ERF domain containing protein, expressed		0.35		3.5		-3.33	
**Plant growth and stress response**											
LOC_Os02g43330		*OsHOX24*		Homeobox associated leucine zipper, putative, expressed		0.12		3.88		-4.98	
LOC_Os03g60570		*OsZFP15*		C2H2 zinc finger protein, expressed		0.77		24.3		-4.98	
LOC_Os02g46030		*OsMyb1R*		MYB family transcription factor, putative, expressed		0.46		7.56		-4.04	
LOC_Os12g03040		*ONAC131*		No apical meristem protein, putative, expressed		0.35		4.91		-3.81	
LOC_Os08g06110		*OsCCA1*		MYB family transcription factor, putative, expressed		37.91		493.14		-3.7	
LOC_Os01g32770				LOB domain-containing protein 40		0.72		9.37		-3.69	
LOC_Os04g43680		*OsMYB4*		MYB family transcription factor, putative, expressed		1.58		19.26		-3.61	
LOC_Os03g60080		*OsNAC9*		NAC domain-containing protein 67, putative, expressed		31.59		350.51		-3.47	
LOC_Os01g48446		*ONAC14*		no apical meristem protein, putative, expressed		2.93		30.49		-3.38	
**Protein synthesis**											
LOC_Os01g60640		*OsWRKY21*		WRKY transcription factor 21		2.33		34.26		-3.88	
LOC_Os01g15900		*OsDof2*		Zinc finger, Dof-type domain containing protein		0.74		9.61		-3.69	
LOC_Os03g53020		*OsbHLH148*		Basic helix-loop-helix transcription factor		5.43		65.63		-3.6	
LOC_Os02g13800		*OsHsfC2a*		HSF-type DNA-binding domain containing protein, expressed		0.55		5.94		-3.43	
LOC_Os02g08440		*OsWRKY71*		WRKY transcription factor 71		55.12		593.57		-3.43	

Numerous phytohormone-related TFs were also downregulated in T8, including ethylene response genes (eight AP2/ERF domain TFs), jasmonate signalling related TFs (four Tify domain TFs and 1 helix-loop-helix DNA binding domain TF) and a further seven DEGs associated with auxin, gibberellin and other phytochemicals (terpenoids and alkaloids) (Fig 2B and S3 Table). A further eight DEGs associated with abiotic/biotic stress responses were downregulated in T8 (Fig 2B and S3 Table). Genes associated to carbon metabolism were found to be downregulated, like *OsINV3* (LOC_Os02g01590), *OsUGlcAE3* (LOC_Os02g54890), sugar/inositol transporter (LOC_Os12g32760) and *OsSWEET2b* (LOC_Os01g50460). Those coding for a glycosyl hydrolase (*OsXTH1*; LOC_Os04g51460), transferase (LOC_Os03g47530) and *OsPCS13* (LOC_Os03g18910) were assigned to cell wall degradation and were downregulated in T8 (Fig 2B).

#### Highly upregulated genes at 16 DAA in response to P-withdrawal

**Fig 3:**
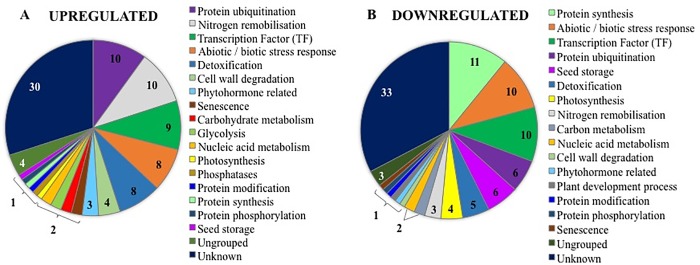
**The 100 most highly upregulated (a) downregulated (b) in T16.** The number in the chart indicates the number of genes that are assigned in the category.

More than 50% of upregulated genes in T16 were grouped into protein ubiquitination, N remobilisation, TFs, detoxification, stress response and cellular hydrolysing processes (Fig 3A). The upregulated genes in T16 were quite distinct from those upregulated in T8, despite both group of plants having been P deprived for the same length of time (i.e. 8 d), which indicates that the responses are influenced by plant development stage. A total of 10 DEGs were annotated to protein ubiquitination, and they were mainly zinc finger domain containing proteins (Fig 3A and S4 Table). Several peptide transporters and an aquaporin protein (*OsTIP4;1*), which are involved in urea transport, were upregulated in addition to four upregulated DEGs associated with releasing ammonium or with the degradation of glutamine, that together were assigned to N remobilisation (Fig 3A).

Nine TFs were upregulated in T16, mostly from the MYB family (Fig 3A and Table 4), which are associated with a range of processes including protein synthesis, protein ubiquitination and plant growth and stress response (Table 4). Eight upregulated DEGs were associated with abiotic/biotic stress response, including universal stress protein (LOC_Os05g28740) and hypothetical protein (Os05g0355450 –no MSU ID matched) that showed 42% identity to the universal stress protein in *Zea mays*. These two genes were highly expressed in T16, with FPKM values of 1,252 and 1,056, respectively (S4 Table). Eight genes coding for proteins involved in detoxification of ROS were upregulated, and these included thioredoxin (TRX), glutaredoxin (*OsGRX23*), peroxidase and FAD dependent oxidoreductase (Fig 3A and S4 Table).

**Table 4:** The expression profile of upregulated transcription factor families in the 100 most differentially expressed genes in T16.

Gene ID	Gene name	Description	FPKM		Log_2_
			Treatment	Control	
**Protein synthesis**					
LOC_Os05g45020	*OsC3H37*	Zinc finger/CCCH transcription factor, putative, expressed	3.64	0.54	2.74
LOC_Os06g15330	*OsCCT20*	CCT/B-box zinc finger protein, putative, expressed	56.37	10.45	2.43
LOC_Os09g36730	*OsMYB108*	MYB family transcription factor, putative, expressed	19.23	2.69	2.84
LOC_Os02g09480		myb-like DNA-binding domain containing protein, putative, expressed	6.24	1.25	2.32
LOC_Os03g55760	*OsKANADI4*	MYB family transcription factor, putative, expressed	5.47	1.04	2.39
**Plant growth and stress response**					
LOC_Os01g74020	*OsPCL12*	MYB family transcription factor, putative, expressed	21.59	1.79	3.6
LOC_Os02g46030	*OsMyb1R*	MYB family transcription factor, putative, expressed	2.24	0.44	2.34
LOC_Os04g49450	*OsMYB511*	MYB family transcription factor, putative, expressed	1.24	0.15	3.01
**Protein ubiquitination**					
LOC_Os12g10660	*OsBBX30*	B-box zinc finger family protein, putative, expressed	2.49	0.43	2.52

Interestingly, two enzymes assigned to carbohydrate metabolism, beta-amylase (LOC_Os10g41550) and beta-fructofuranosidase (invertase: LOC_Os09g08120), were upregulated in T16 (Fig 3A and S4 Table). Similarly, four DEGs assigned to cell wall degradation, including two glycosyl hydrolases (*OsXTH15* [LOC_Os06g22919 and LOC_Os05g31140]) and glycosyltransferase (LOC_Os08g38710), were upregulated in T16, which indicates that xyloglycan was catalysed, probably corresponding to cell wall degradation (Fig 3A and S4 Table). In contrast to the nine photosynthesis related genes upregulated in T8, only one photosystem II 10 kDa polypeptide (LOC_Os07g05365) was upregulated in T16, and one phosphatase involved in phosphoryl transfer (LOC_Os03g49440) was upregulated (Fig 3A).

#### Highly downregulated genes at 16 DAA in response to P-withdrawal

Eleven of the downregulated DGEs in T16 were assigned to protein synthesis and nine out of eleven were specifically related to heat shock protein (Hsp) (S4 Table). In addition, three TFs in the HSF (heat shock transcription factor) family were also downregulated (Fig 3B and Table 5). As occurred in T8, several ERF family TFs that are involved in ethylene response and a bHLH family TF that is involved in jasmonate signalling were also downregulated in T16 (Table 5). Ten DEGs assigned to abiotic/biotic stress were also downregulated in T16 (Fig 3B and S4 Table).

**Table 5:** The expression profile of downregulated transcription factor families in the 100 most differentially expressed genes in T16.

	Gene name	Description	FPKM		Log_2_
			Treatment	Control	
**Ethylene response**					
LOC_Os09g35020	*OsERF133*	AP2 domain containing protein, expressed	0.25	3.76	-3.9
LOC_Os08g36920	*OsERF104*	AP2 domain containing protein, expressed	0.10	1.3	-3.74
LOC_Os09g35010	*OsERF31*	Dehydration-responsive element-binding protein, putative, expressed	2.76	24.96	-3.18
LOC_Os04g52090	*OsERF77*	AP2 domain containing protein, expressed	4.97	31.43	-2.66
**Jasmonate signalling**					
LOC_Os04g23550	*OsbHLH006*	Basic helix-loop-helix family protein, putative, expressed	0.14	0.78	-2.49
**Protein ubiquitination**					
LOC_Os09g26210		C2H2 zinc finger protein, expressed	1.49	8.75	-2.55
**Protein synthesis**					
LOC_Os09g35790	*OsHsfB2c*	HSF-type DNA-binding domain containing protein, expressed	0.28	21.38	-6.23
LOC_Os08g43334	*OsHsfB2b*	HSF-type DNA-binding domain containing protein, expressed	0.13	3.93	-4.97
LOC_Os04g48030	*OsHsfB2a*	Heat stress transcription factor B-1, putative, expressed	0.5	3.2	-2.68
**Plant growth and stress response**					
LOC_Os01g64310	*ONAC59*	No apical meristem protein, putative, expressed	0.22	2.25	-3.38

Four DEGs involved in photosynthesis were downregulated, three of which were early light-induced proteins (LOC_Os01g14410, LOC_Os07g08150, LOC_Os07g08160; Fig 3B and S4 Table).

Notably, six genes associated with seed storage proteins, including glutelin and cupin (LOC_Os03g31360, LOC_Os01g55690, LOC_Os02g25640, LOC_Os06g09820, LOC_Os07g38630, LOC_Os06g03390), were downregulated in T16. *OsGRX2*, a glutaredoxin, was downregulated, in addition to a cytosolic ascorbate peroxidase (*OsAPx2*), an oxidoreductase (*OsAKR2*), a thioredoxin protein (LOC_Os02g56900) and heavy metal-associated domain (LOC_Os10g38870) that are involved in detoxification processes (Fig 3B). The downregulation of the glucose-6-phosphate/phosphate translocator, *OsGPT2-3* (LOC_Os07g33910) and the sucrose transporter *OsSWEET13* (LOC_Os12g29220) indicates the downregulation of carbon metabolism in T16 (Fig 3B).

### Expression of Pi transporters in flag leaves during grain filling

**Fig 4:**
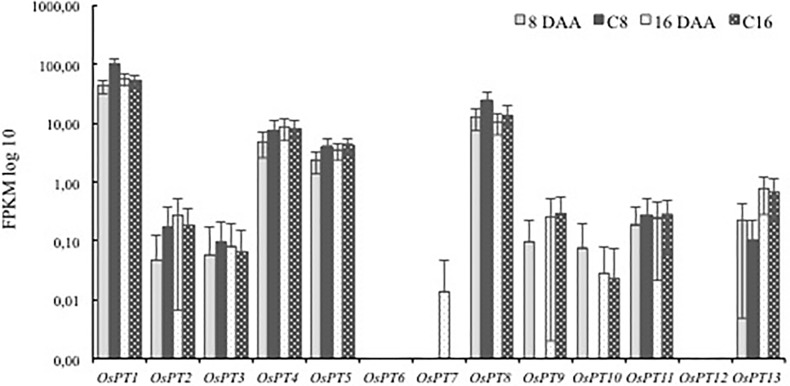
The expression of the PHT 1 family of rice P transporters in rice flag leaves during grain filling. P values *** < 0.001, no marks = non-significant.

It has been well documented that Pi transporter family 1 (Pht 1; *OsPT* 1–13) genes are involved in Pi uptake and distribution [12, 34], so the expression of these genes was examined in flag leaves during grain filling. Only four OsPT genes (*OsPT1*, *OsPT4*, *OsPT5* and *OsPT8*) were expressed at detectable levels (i.e. FPKM > 1). The expression of *OsPT1* was the highest in both treatments at both time points (FPKM > 40), followed by *OsPT8* (FPKM > 10), while *OsPT4* and *OsPT5* had lower expression levels (FPKM < 10) (Fig 4). Notably, *OsPT1*, *OsPT4*, *OsPT5* and *OsPT8* were downregulated in T8, whereas they were not differentially expressed at 16 DAA (Fig 4).

## Discussion

While the response of young plants to P deprivation is well documented at the physiological and molecular levels, little is known about the physiological and molecular regulation of P mobilisation from senescing leaves during grain filling. We previously identified several genes that showed expression profiles consistent with a role in P remobilisation from senescing rice flag leaves, but in the absence of any specific P treatment, it was not possible to definitively conclude their involvement in the liberation and transport of P from leaves during grain filling [10]. While the treatments imposed in the current study involved deprivation of P for 8 d (either from anthesis to 8 DAA or from 8 DAA to 16 DAA), the absence of any consistent upregulation of the 11 key PSR genes in T8 or T16 clearly demonstrates that the response observed was distinct from the typical P deprivation response observed in younger plants under P starvation (Table 1) and by the same token, emphasising the different metabolic states present in young and mature plants.

### Different response to P withdrawal depending on grain filling stage

Not only did the withdrawal of P from the nutrient solution during grain filling elicit a different transcriptional response to that from young plants, but a distinct difference between the response to 8 d of P deprivation during the early stage of grain filling (8 DAA) and the response to 8 d of P deprivation at later stage (16 DAA) was observed. At 8 DAA, a total of 34 upregulated DEGs assigned to nucleic acid metabolism and photosynthesis were upregulated, while at 16 DAA, only two nucleic acid metabolism genes were upregulated, and four photosynthesis related genes were actually downregulated in T16 (Figs 2 and 3). Downregulation of photosynthesis related genes is in accordance with the photosynthetic rate data from these plants—reported in Jeong *et al*. [22]—where the withdrawal of P supply from anthesis to 8 DAA did not significantly reduce photosynthesis activity compared to the control plants (14.5 μmol CO_2_ m^-2^ s^-1^), while withdrawal of P supply at 8 DAA significantly reduced this activity to 7.5 μmol CO_2_ m^-2^ s^-1^ at 16 DAA compared to the control plants (14.8 μmol CO_2_ m^-2^ s^-1^) (S1 Table). However, it is possible that the response observed is specific to the cultivar studied (cv. IR64) and further research is needed to determine whether this response is observed in a wider range of rice cultivars.

### Response to P withdrawal at early stage of grain filling (8DAA)

**Fig 5:**
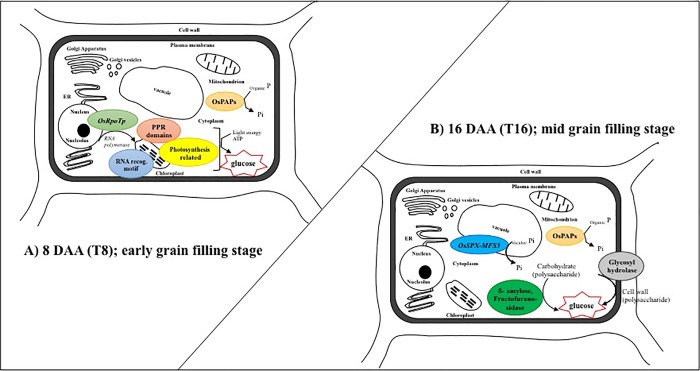
Hypothesised response to P withdrawal at the cellular level during grain filling (A) at 8 DAA and (B) 16 DAA based on differentially expressed genes.

At 8DAA, PPR proteins and RNA polymerase (*OsRpoTp*) were highly upregulated, indicating activation of RNA metabolic processes in the chloroplast [35–38], that together wth the upregulation of the whirly TF (LOC_Os06g05350) (S3 Table), indicate the correct functioning of chloroplasts in T8 [39]. We also observed significant upregulation of photosystem II (PSII) oxygen evolving complex protein PsbQ family protein which stabilises PsbP binding, thereby contributing to the maintenance of the catalytic manganese (Mn) cluster of the water oxidation machinery [40]. In addition, the significant upregulation of an aldose 1-epimerase family protein (AEP; LOC_Os03g53710), indicated the activating of energy metabolism, since AEP participates in the conversion of D-galactose to D-glucose 6-phosphate by either the Leloir pathway or the pyrophosphorylase pathway [41]. This pathway is required since galactose itself cannot be used for glycolysis directly. Taking these observations together, it appears that the deprivation of P from anthesis to 8 DAA in plants that received adequate P supply during vegetative growth induced a plant transcriptional response aimed at sustaining photosynthesis by upregulation of genes directly involved in chloroplast and thylakoid functioning. This would enable continued production of carbon substrates to be used in glycolysis and the TCA cycle for sustained growth and to meet the carbon demands of the developing grains (Fig 5). The most notable changes in gene expression among the 32 PRGs (those identified in the study of Jeong et al. [10]) were the upregulation of three OsPAP genes (*OsPAP15*, *OsPAP10a* and *OsPAP1d*) and downregulation of *OsSPX-MFS1* and *OsSPX-MFS3* (Table 2). Given that OsPAP genes are known to play a role in liberating P from organic P sources [17, 30, 42, 43], and *OsSPX-MFS1* and *OsSPX-MFS3* function as phosphate influx and efflux transporters in rice, respectively [20, 21], it is possible that P liberated from organic P sources was sufficient to meet the competing P demands of developing grains and concurrent leaf photosynthetic processes without requiring vacuolar P. Notably, the P demand of developing grains is relatively low during this period; most P is loaded into grains from around 6 DAA to 15 DAA in a sigmodal accumulation pattern [9, 13], and hence, only a small proportion is accumulated in the grains by 8 DAA. The downregulation of both *OsSPX-MFS1* and *OsSPX-MFS3* supports the notion that plants had sufficient cytoplasmic P without the need to remove/use any P stored in the vacuole (Table 2). Regardless, the data clearly support results from our earlier study [10] that implicated *OsPAP15*, *OsPAP10a* and *OsPAP1d* in the mobilisation of P from senescing leaves.

### Response to P withdrawal from 8 to 16 days after anthesis

**Fig 6:**
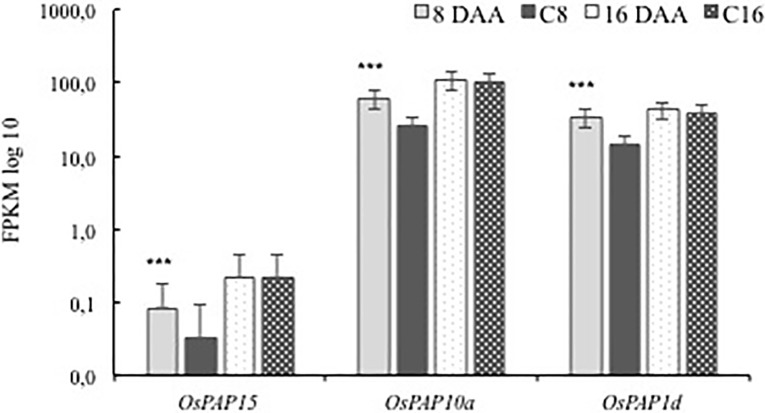
The expression of three OsPAPs in rice flag leaves during grain filling. P values *** < 0.001, no marks = non-significant.

In contrast to the response from plants to P deprivation between anthesis and 8 DAA (T8), the overall response of plants to P deprivation between 8 DAA and 16 DAA (T16) appeared to be accelerated leaf senescence. This involved downregulation of photosynthesis-related genes including early light induced proteins (LOC_Os07g08150, LOC_Os07g08160 and LOC_Os01g14410) and *OsPsbS2* which is a light harvesting complex gene within PSII [44]. Parallel to these changes, the upregulation of polysaccharide degradation associated genes such as beta-amylase (*OsBAM5*), beta-fructofuranosidase (*OsCIN8*) for degrading of storage polysaccharides, and two glycosyl hydrolases (defensin *OsDEFL9*) and a glycosyltransferase (LOC_Os08g38710) for degrading of structural polysaccharide (cell wall) were also observed (Fig 5 and S4 Table). The large number of upregulated DEGs assigned to protein ubiquitination and N remobilisation that indicated an increase in protein degradation [45], was also consistent with accelerated leaf senescence in T16. Interestingly, the three OsPAP genes that were upregulated in T8 also showed high expression levels in the control plants at 16 DAA (Fig 6), suggesting that some degree of leaf senescence had already been initiated in these plants.

*OsSPX-MFS3*, which plays a role in the efflux of P from the vacuole to maintain a constant cytoplasmic P level [21] (Fig 5), was upregulated in T16 (Table 2). This may suggest that plants were using vacuolar P stores to meet a critical P deficit in cells to maintain their function, or to meet the high P demands of developing grains, however, leaf P fraction data from these plants indicated no difference in flag leaf inorganic P (Pi) content between T16 plants and control plants [22]. This anomaly may be due to a lack of resolution in the leaf P fraction data due to inherent variability among biological replicates or in the methods used for fractionation. The expression pattern of *OsSPX-MFS3*, however, is indicative of a role in P mobilisation from senescing leaves, and further study of the function of this gene during grain filling is warranted.

Phosphorus is remobilised from old tissues to young tissues via the phloem [46], predominantly as Pi [47]. Transport of Pi into the phloem is thought to occur mostly through the action of members of the PHT1 family of transporters. In rice, six PHT1 family transporters (*OsPT1*, *OsPT2*, *OsPT6*, *OsPT8*, *OsPT9* and *OsPT10*) have been shown to play a role in P remobilisation in the shoots of young plants [48–51], but the function of these transporters during leaf senescence has not been fully resolved [11]. The absence of any upregulation of PHT family transporters in T8 or T16 plants may suggest that their induction is not necessary and that the basal activity present in control plants is enough to satisfy Pi transport along the phloem.

A recent study also revealed that a node specific transporter, sulfate transporter-like phosphorus distribution transporter (*SPDT*) plays an important role in P remobilisation from old tissues to young tissues through nodes in rice, with a 20% decrease in grain P content observed in grains of an *spdt* mutant [52]. No differential expression of *SPDT* was found in our data since *SPDT* is a gene specifically expressed in nodes (S3 Fig). Similar reductions in grain P concentration were observed in rice mutants where the sulfate transporter *OsSULTR3;3* was knocked out [53]. While *SPDT* and *OsSULTR3;3* provide potential targets for manipulation of grain P content, resolving the role of three OsPAPs (*OsPAP1d*, *OsPAP10a* and *OsPAP15*) and *OsSPX-MFS3* in flag leaves during grain filling may lead to additional targets for manipulation of genes to reduce P concentrations in the grains of rice.

## Supporting information

S1 Fig Schematic diagram of experimental design and timing of permanent P withdrawal from the nutrient solution. (PDF)

S2 Fig The expression of four OsSPX-MFSs in rice flag leaves during grain filling. Statistical analysis was calculated individually by the sample T8 vs C8, T16 vs C16. P values *** < 0.001, no marks = non-significant.
(PDF)

S3 Fig The expression of SPDT in rice flag leaves during grain filling. Statistical analysis was calculated individually by the sample T8 vs C8, T16 vs C16. P values *** < 0.001, no marks = non-significant.
(PDF)

S1 Table Concentration of total phosphorus and phosphorus fractions in flag leaves, and photosynthetic rate in sampled flag leaves. (PDF)

S2 Table Sequencing reads and mapping statistics. (PDF)

S3 Table The list of 100 of most each up and downregulated differential expressed genes in T8. (PDF)

S4 Table The list of 100 of most each up and downregulated differential expressed genes in T16. (PDF)

S5 Table 32 PRGs analysed in the present study. (PDF)
