# Supplementary figures and images for: Transcriptional response of rice flag leaves to restricted external phosphorus supply during grain filling in rice cv. IR64
Source: PLoS One. 2018 Sep 13;13(9):e0203654. doi: 10.1371/journal.pone.0203654 (PMC6136725; doi:10.1371/journal.pone.0203654)

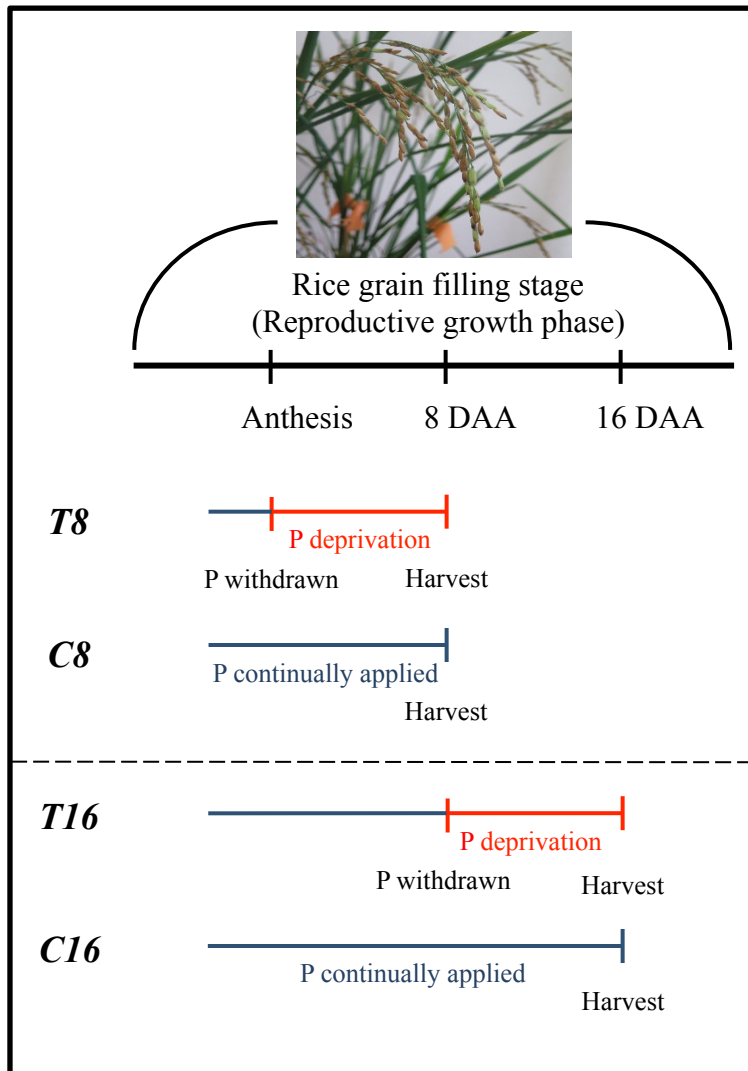

**S1 Fig.**

Supplement: S1 Fig — (PDF) [file pone.0203654.s001.pdf]

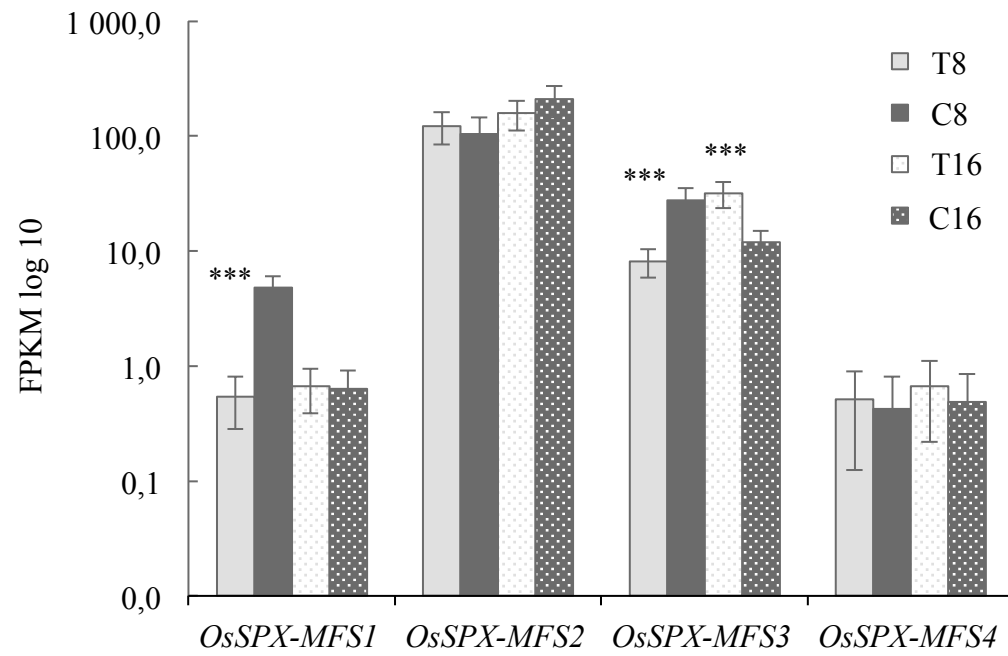

**S2 Fig.**

Supplement: S2 Fig — Statistical analysis was calculated individually by the sample T8 vs C8, T16 vs C16. P values *** < 0.001, no marks = non-significant. (PDF) [file pone.0203654.s002.pdf]

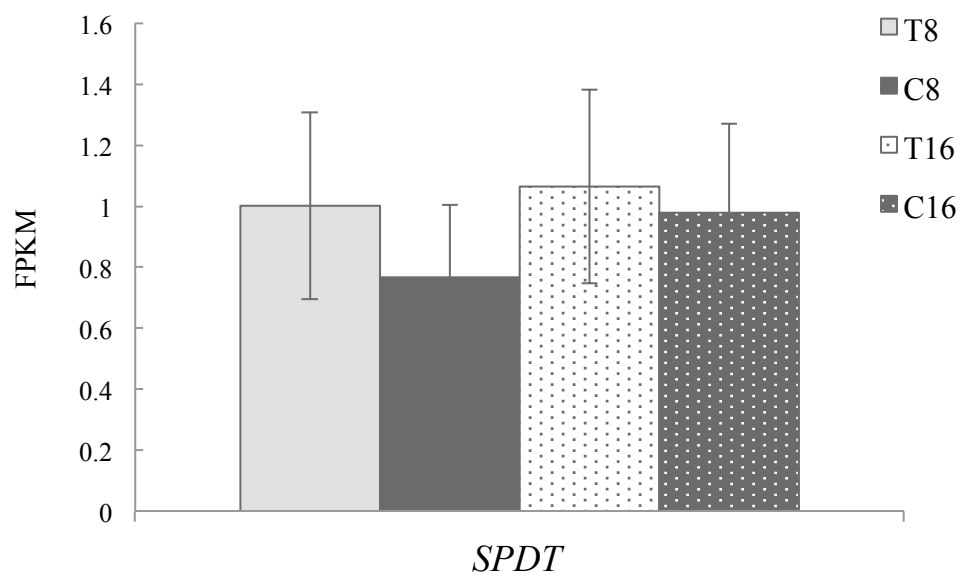

**S3 Fig.**

Supplement: S3 Fig — Statistical analysis was calculated individually by the sample T8 vs C8, T16 vs C16. P values *** < 0.001, no marks = non-significant. (PDF) [file pone.0203654.s003.pdf]
