# Supplementary material for: Transcriptional response of rice flag leaves to restricted external phosphorus supply during grain filling in rice cv. IR64
Source: PLoS One. 2018 Sep 13;13(9):e0203654. doi: 10.1371/journal.pone.0203654 (PMC6136725; doi:10.1371/journal.pone.0203654)
Supplement: S1 Table — (PDF) [file pone.0203654.s004.pdf]

Supplementary Table S1. Concentration of total phosphorus and phosphorus fractions in flag leaves, and photosynthetic rate in sampled flag leaves.

	T8	C8	T16	C16
Total P concentration (mg g^{-1})	1.13±0.02 [a*]	1.26±0.09 [a]	1.04±0.03 [a]	1.15±0.06 [a]
Lipid P concentration (mg g^{-1})	0.35±0.03 [a]	0.44±0.03 [a]	0.27±0.01 [a]	0.35±0.03 [a]
Metabolic P concentration (mg g^{-1})	0.18±0.01 [a]	0.17±0.02 [a]	0.10±0.00 [a]	0.14±0.02 [a]
Nucleic P concentration (mg g^{-1})	0.41±0.03 [a]	0.37±0.05 [a]	0.38±0.01 [a]	0.39±0.02 [a]
Residue P concentration (mg g^{-1})	0.07±0.01 [a]	0.06±0.01 [a]	0.06±0.00 [a]	0.06±0.00 [a]
Inorganic P concentration (mg g^{-1})	0.20±0.01 [a]	0.21±0.01 [a]	0.19±0.05 [a]	0.20±0.00 [a]
Photosynthetic rate (μmol $CO_2 m^{-2} s^{-1}$)	14.11±0.5 [a]	14.55±0.3 [a]	7.49±0.5 [b]	14.83±0.6 [a]

* Statistical analysis was calculated individually by the sample T8 vs C8, T16 vs C16, means that do not share a common letter are significantly different at $P < 0.05$ (n=3).
