# Supplementary material for: Transcriptional response of rice flag leaves to restricted external phosphorus supply during grain filling in rice cv. IR64
Source: PLoS One. 2018 Sep 13;13(9):e0203654. doi: 10.1371/journal.pone.0203654 (PMC6136725; doi:10.1371/journal.pone.0203654)
Supplement: S2 Table — (PDF) [file pone.0203654.s005.pdf]

Supplementary Table S2. Sequencing reads and mapping statistics.

Samples	Replications	Raw reads	High quality reads		Mapping to genome	
			Number	%	Number	%
T8	1	24,854,023	23,977,352	96.47	21,052,810	87.80
	2	25,304,583	24,444,144	96.60	21,426,497	87.65
	3	25,526,544	24,653,639	96.28	21,604,784	87.63
C8	1	22,487,598	21,650,648	96.28	18976393	87.64
	2	25,340,868	24,457,345	96.51	21,670,356	88.60
	3	32,176,783	31,085,803	96.61	27,431,124	88.24
T16	1	27,276,116	26,184,472	96.00	23,108,395	88.25
	2	24,282,172	23,454,736	96.59	20,119,329	85.77
	3	25,581,871	24,736,233	96.69	21,661,078	87.56
C16	1	24,758,236	23,928,383	96.65	20,960,512	87.59
	2	22,766,265	22,006,105	96.66	19,266,214	87.54
	3	23,669,273	22,796,510	96.31	19,934,157	87.44
