# Supplementary material for: Transcriptional response of rice flag leaves to restricted external phosphorus supply during grain filling in rice cv. IR64
Source: PLoS One. 2018 Sep 13;13(9):e0203654. doi: 10.1371/journal.pone.0203654 (PMC6136725; doi:10.1371/journal.pone.0203654)
Supplement: S3 Table — (PDF) [file pone.0203654.s006.pdf]

Supplementary Table S3. The list of 100 of most each up and downregulated differential expressed genes in T8.

Gene ID	MSU ID	Gene name	T8	C8	Log_2	Description	Classification
**100 UP regulated in T8**							
Os03g0725400	LOC_Os03g51550	*OsWD40-86*	1.3	0.1	3.7	WD repeat-containing protein 5, putative, expressed	Abiotic / biotic stress related
Os03g0853200	LOC_Os03g63620	*OsTET6*	1.3	0.2	3.0	Tetraspanin domain containing protein	Abiotic / biotic stress related
Os08g0436500	LOC_Os08g33890		2.9	0.4	2.7	Hypothetical conserved gene	Abiotic / biotic stress related
Os01g0814800	LOC_Os01g59920		4.1	0.5	3.1	cysteine synthase, chloroplast precursor, putative, expressed	Amino acid metabolism
Os08g0342400	LOC_Os08g25390		2.8	0.3	3.1	bifunctional aspartokinase/homoserine dehydrogenase, chloroplast precursor, putative, expressed	Amino acid metabolism
Os04g0610500	LOC_Os04g52100		16.1	2.7	2.6	peptidase, M24 family protein, putative, expressed	Amino acid metabolism
Os03g0808150	LOC_Os03g59330		4.3	0.6	2.9	polygalacturonase, putative, expressed	Cell wall degradation
Os07g0684100	LOC_Os07g48510		4.8	0.2	4.6	Thioredoxin, putative, expressed	Detoxification
Os07g0529600	LOC_Os07g34570	*OsDR8, OsXNP*	4340.1	361.6	3.6	FAD dependent oxidoreductase domain containing protein, expressed	Detoxification
Os01g0829000	LOC_Os01g61320		8.9	0.8	3.5	Thioredoxin-like fold domain containing protein	Detoxification
Os02g0774100	LOC_Os02g53400		11.3	1.6	2.8	Similar to thioredoxin-like 5	Detoxification
Os06g0115400	LOC_Os06g02500		25.7	3.8	2.8	Superoxide dismutase, chloroplast, putative, expressed	Detoxification
Os03g0602600	LOC_Os03g40550		14.1	1.7	3.1	pfkB family, putative, expressed	Glycolysis
Os04g0445700	LOC_Os04g36800		4.6	0.5	3.1	3-oxoacyl-synthase, putative, expressed	Lipid synthesis
Os09g0277800	LOC_Os09g10600		1.0	0.1	2.8	enoyl-acyl-carrier-protein reductase NADH, chloroplast precursor, expressed	Lipid synthesis
Os01g0872000	LOC_Os01g65150		1.6	0.1	4.0	proton-dependent oligopeptide transport, putative, expressed	Nitrogen remobilisation

Supplementary Table S3. Continued.

Gene ID	MSU ID	Gene name	T8	C8	Log_2	Description	Classification
Os08g0248800	LOC_Os08g15030		5.4	0.8	2.8	Similar to Aspartate carbamoyltransferase 3, chloroplast precursor	Nitrogen remobilisation
Os05g0231700	LOC_Os05g14240	*OsTIP4;1*	57.3	8.7	2.7	aquaporin protein, putative, expressed	Nitrogen remobilisation
Os01g0871900	LOC_Os01g65140		0.9	0.1	2.7	peptide transporter PTR2, putative, expressed	Nitrogen remobilisation
Os01g0702000	LOC_Os01g50622	*OsBBD1*	20.0	0.8	4.6	Protein of unknown function DUF151 domain containing protein	Nucleic acid metabolism
Os09g0482680	LOC_Os09g30466		11.2	0.7	3.9	Nuclear ribonuclease Z, putative, expressed	Nucleic acid metabolism
Os12g0493900	LOC_Os12g31000		2.5	0.2	3.9	pumilio-family RNA binding repeat domain containing protein, expressed	Nucleic acid metabolism
Os03g0765400	LOC_Os03g55660, LOC_Os03g55670		5.4	0.5	3.5	nucleoporin, putative, expressed	Nucleic acid metabolism
Os01g0901900	LOC_Os01g67570		14.4	1.5	3.3	S1, RNA binding domain containing protein	Nucleic acid metabolism
Os03g0216300	LOC_Os03g11690		2.2	0.3	3.1	Pentatricopeptide repeat domain containing protein	Nucleic acid metabolism
Os05g0551900	LOC_Os05g47850		17.3	2.2	3.0	Similar to EMB1865 (embryo defective 1865); RNA binding	Nucleic acid metabolism
Os07g0615000	LOC_Os07g42354		2.3	0.3	2.9	Similar to PPR repeat domain containing protein	Nucleic acid metabolism
Os01g0306800	LOC_Os01g20110		13.5	1.8	2.9	Conserved hypothetical protein	Nucleic acid metabolism
Os05g0574800	LOC_Os05g49920		13.6	1.9	2.9	pentatricopeptide, putative, expressed	Nucleic acid metabolism
Os06g0683500	LOC_Os06g46960		1.1	0.1	2.8	Similar to predicted protein	Nucleic acid metabolism
Os05g0566300	LOC_Os05g49130		5.1	0.7	2.8	16S rRNA processing protein RimM containing protein, expressed	Nucleic acid metabolism
Os03g0736400	LOC_Os03g52640		30.4	4.5	2.8	Protein of unknown function methylase putative domain containing protein	Nucleic acid metabolism
Os01g0916600	LOC_Os01g68790	*OsGRP1*	45.1	6.7	2.8	RNA recognition motif, glycine rich protein (GRP) domain containing protein	Nucleic acid metabolism
Os11g0679900	LOC_Os11g45410		1.1	0.2	2.7	Conserved hypothetical protein	Nucleic acid metabolism

Supplementary Table S3. Continued.

Gene ID	MSU ID	Gene name	T8	C8	Log_2	Description	Classification
Os09g0279500	LOC_Os09g10760		55.7	8.9	2.6	RNA recognition motif containing protein, putative, expressed	Nucleic acid metabolism
Os03g0734000	LOC_Os03g52360		0.8	0.1	2.6	Pentatricopeptide repeat containing protein	Nucleic acid metabolism
Os03g0812000	LOC_Os03g59750		7.0	1.1	2.6	DNA topoisomerase, type IIA, subunit A or C-terminal domain containing protein	Nucleic acid metabolism
Os03g0824100	LOC_Os03g60910		43.3	7.1	2.6	PPR repeat domain containing protein, putative, expressed	Nucleic acid metabolism
Os06g0332600	LOC_Os06g22660		0.9	0.1	2.6	RNA recognition motif, glycine rich protein (GRP) domain containing protein	Nucleic acid metabolism
Os03g0376600	LOC_Os03g25960		24.5	4.0	2.6	RNA recognition motif, glycine rich protein (GRP) domain containing protein	Nucleic acid metabolism
Os06g0652000	LOC_Os06g44230	*OsRpoTp*	13.4	2.2	2.6	DNA-directed RNA polymerase 3A, chloroplast precursor, putative, expressed	Nucleic acid metabolism
Os11g0131200	LOC_Os11g03670		2.3	0.4	2.6	Similar to Mpv17/PMP22 family protein, expressed	Nucleic acid metabolism
Os06g0168600	LOC_Os06g07210	*RNRL1*	0.8	0.1	2.6	Ribonucleotide reductase, Chloroplast biogenesis	Nucleic acid metabolism
Os08g0191900	LOC_Os08g09270		17.2	2.9	2.6	pentatricopeptide, putative, expressed, Pentatricopeptide repeat domain containing protein	Nucleic acid metabolism
Os07g0489800	LOC_Os07g30670		17.8	1.1	4.0	Ferredoxin-type domain containing protein	Photosynthesis
Os08g0344600	LOC_Os08g25624		13.7	1.2	3.5	Similar to Triose phosphate/phosphate translocator, non-green plastid, chloroplast precursor (CTPT).	Photosynthesis
Os08g0139100	LOC_Os08g04450		266.9	24.2	3.5	Similar to DAG protein, chloroplast precursor	Photosynthesis
Os10g0462800	LOC_Os10g32540	*pTAC3*	20.2	2.1	3.3	Similar to PTAC3 (PLASTID TRANSCRIPTIONALLY ACTIVE3); DNA binding / nucleic acid binding	Photosynthesis
Os02g0773500	LOC_Os02g53350		67.9	7.2	3.2	Protein of unknown function DUF3464 domain containing protein	Photosynthesis

Supplementary Table S3. Continued.

Gene ID	MSU ID	Gene name	T8	C8	Log_2	Description	Classification
Os03g0748300	LOC_Os03g53710		50.0	5.8	3.1	Aldose 1-epimerase family protein	Photosynthesis
Os09g0439500	LOC_Os09g26810		11.6	1.5	3.0	Similar to Type II chlorophyll a/b binding protein from photosystem I precursor	Photosynthesis
Os02g0578400	LOC_Os02g36850		10.2	1.7	2.6	Photosystem II oxygen evolving complex protein PsbQ family protein	Photosynthesis
Os10g0533100	LOC_Os10g38910		85.6	14.5	2.6	protein PAM68, chloroplastic	Photosynthesis
Os04g0223500	LOC_Os04g14690		8.0	0.8	3.2	Dimethylaniline monooxygenase, N-oxide-forming domain containing protein	Phytohormone related
Os10g0439924	LOC_Os10g30410	*Oscyp71Z8*	1.7	0.2	3.2	Cytochrome P450 family protein	Phytohormone related
Os12g0256000	LOC_Os12g15400		1.9	0.3	2.8	Alpha/beta hydrolase fold-3 domain containing protein	Phytohormone related
Os04g0616600	LOC_Os04g52630		0.9	0.1	2.8	Serine/threonine protein kinase-related domain containing protein	Protein phosphorylation
Os09g0382400	LOC_Os09g21460		5.8	0.9	2.7	expressed protein	Protein phosphorylation
Os04g0675400	LOC_Os04g57880	*OsDjC45*	10.5	0.4	4.6	heat shock protein DnaJ, putative, expressed	Protein synthesis
Os12g0277500	LOC_Os12g17910		128.1	20.8	2.6	T-complex protein, putative, expressed	Protein synthesis
Os12g0178700	LOC_Os12g07880	*OsARC5*	1.1	0.1	3.4	Similar to ARC5 (ACCUMULATION AND REPLICATION OF CHLOROPLAST 5); GTP binding / GTPase	Protein translation
Os11g0116400	LOC_Os11g02450		5.9	0.7	3.1	Similar to Elongation factor P	Protein translation
Os12g0563200	LOC_Os12g37610		31.2	4.5	2.8	ribosomal protein S6, putative, expressed	Protein translation
Os03g0781000	LOC_Os03g56840		12.7	2.1	2.6	GTP-binding protein, putative, expressed	Protein translation
Os05g0568300	LOC_Os05g49320		51.3	8.7	2.6	ribosomal protein L7/L12 C-terminal domain containing protein, expressed	Protein translation
Os05g0478000	LOC_Os05g40020		2.6	0.2	3.4	RING/FYVE/PHD-type domain containing protein	Protein ubiquitination

Supplementary Table S3. Continued.

Gene ID	MSU ID	Gene name	T8	C8	Log_2	Description	Classification
Os02g0249200	LOC_Os02g15110		13.9	2.3	2.6	zinc finger, C3HC4 type domain containing protein, expressed	Protein ubiquitination
Os10g0445400	LOC_Os10g30850	*OsHCI1*	9.7	1.6	2.6	Zinc finger, RING-type, putative, expressed	Protein ubiquitination
Os01g0922700	LOC_Os01g69840		170.6	4.4	5.3	Conserved hypothetical protein	Senescence
Os08g0260600	LOC_Os08g16050	*OsTET11*	5.4	0.3	4.0	Tetraspanin domain containing protein	Senescence
Os09g0287000	LOC_Os09g11480	*OsSub1B*	13.8	0.6	4.4	AP2 domain containing protein, expressed	TF-ERF family; Ethylene response factor
Os06g0145800	LOC_Os06g05350		1.7	0.2	2.8	whirly transcription factor domain containing protein, expressed	TF-Whirly Family; photosynthesis
Os03g0758900	LOC_Os03g55164	*OsWRKY4, OsWRKY122*	19.7	3.0	2.7	WRKY transcription factor 4	TF-WRKY Family; Protein systhesis
Os01g0143800	LOC_Os01g05060		65.8	4.8	3.8	Mitochondrial glycoprotein family protein.	Ungrouped
Os02g0269200	LOC_Os02g16909		2.2	0.2	3.4	Dynein light chain, type 1 family protein	Ungrouped
Os08g0508500	LOC_Os08g39810		7.0	0.8	3.2	Similar to predicted protein	Ungrouped
Os06g0671700	LOC_Os06g45980		10.8	1.4	2.9	toprim domain-containing protein, putative, expressed	Ungrouped
Os01g0268900	LOC_Os01g16340		3.1	0.4	2.8	Conserved hypothetical protein	Ungrouped
Os11g0546500	LOC_Os11g34390	*OsGT6*	1.2	0.2	2.8	glycosyltransferase, putative, expressed	Ungrouped
Os02g0827600	LOC_Os02g58120		6.6	1.0	2.8	Protein of unknown function DUF3531 domain containing protein	Ungrouped
Os10g0547650			1.2	0.1	4.1	Hypothetical protein	Unknown
Os02g0674233	LOC_Os02g45225		2.0	0.1	4.1	Hypothetical conserved gene	Unknown
Os03g0725601			2.4	0.2	4.0	Non-protein coding transcript	Unknown
Os05g0551801			13.2	1.4	3.3	Hypothetical gene	Unknown
Os01g0101175			2.1	0.2	3.1	Non-protein coding transcript	Unknown
Os11g0594700	LOC_Os11g38210		4.2	0.5	3.0	Protein of unknown function DUF538 family protein	Unknown
Os10g0439986			5.7	0.7	3.0	Hypothetical protein	Unknown

Supplementary Table S3. Continued.

Gene ID	MSU ID	Gene name	T8	C8	Log_2	Description	Classification
Os09g0517825			1.4	0.2	2.9	Hypothetical protein	Unknown
Os07g0223001			13.0	1.7	2.9	Non-protein coding transcript	Unknown
Os01g0901950			22.0	2.9	2.9	Non-protein coding transcript	Unknown
Os06g0263801			3.5	0.5	2.9	Hypothetical gene	Unknown
Os04g0438300	LOC_Os04g35760		6.4	0.9	2.8	Uncharacterised protein family UPF0090 domain containing protein	Unknown
Os03g0656900	LOC_Os03g45400		55.6	7.8	2.8	NusB/RsmB/TIM44 domain containing protein	Unknown
Os03g0567100	LOC_Os03g36960		2.9	0.4	2.8	expressed protein	Unknown
Os06g0536200			2.3	0.3	2.8	Hypothetical gene	Unknown
Os05g0565400	LOC_Os05g49060		34.8	5.2	2.7	Protein of unknown function DUF561 family protein	Unknown
Os03g0821275			11.7	1.8	2.7	Non-protein coding transcript	Unknown
Os02g0531450	LOC_Os02g32940		1.9	0.3	2.7	Conserved hypothetical protein	Unknown
Os10g0154600	LOC_Os10g06610		6.8	1.1	2.6	Conserved hypothetical protein	Unknown
Os10g0522700	LOC_Os10g37860		8.8	1.4	2.6	Conserved hypothetical protein	Unknown

100 DOWN regulated in T8

Os02g0165100	LOC_Os02g06930	*OsRLCK63*	0.4	4.1	-3.3	protein kinase, putative, expressed	Abiotic / biotic stress response
Os01g0314800	LOC_Os01g21250	*OsLEA9*	61.6	1070.6	-4.1	Late embryogenesis abundant protein 3 family protein	Abiotic / biotic stress response
Os04g0684900	LOC_Os04g58810	*OsCAF1B*	6.2	106.0	-4.1	CAF1 family ribonuclease containing protein, putative, expressed	Abiotic / biotic stress response
Os01g0855600	LOC_Os01g63690	*HS1*	0.1	1.4	-4.1	hs1, putative, expressed	Abiotic / biotic stress response
Os03g0676400	LOC_Os03g47280	*OsVQ13*	0.4	6.7	-3.9	VQ domain containing protein	Abiotic / biotic stress response

Supplementary Table S3. Continued.

Gene ID	MSU ID	Gene name	T8	C8	Log_2	Description	Classification
Os01g0864500	LOC_Os01g64470	*OsSDS1*	4.1	60.0	-3.9	Harpin-induced 1 domain containing protein	Abiotic / biotic stress response
Os05g0161500	LOC_Os05g06920		5.4	76.6	-3.8	RelA/SpoT domain containing protein	Abiotic / biotic stress response
Os01g0278000	LOC_Os01g17050	*OsVQ1*	4.2	44.2	-3.4	VQ domain containing protein	Abiotic / biotic stress response
Os03g0729800	LOC_Os03g51990	*OsSTA113*	0.1	1.0	-3.8	ACT domain containing protein, expressed	Amino acid metabolism
Os02g0106100	LOC_Os02g01590	*OsINV3, OsVIN2*	0.5	48.6	-6.7	Similar to Fructosyltransferase, glycosyl hydrolases,	Carbon metabolism
Os02g0791500	LOC_Os02g54890	*OsUGlcAE3*	3.1	44.6	-3.8	Similar to Nucleotide sugar epimerase-like protein (UDP-D-glucuronate 4- epimerase)	Carbon metabolism
Os12g0512100	LOC_Os12g32760		0.3	4.2	-3.8	Sugar/inositol transporter domain containing protein	Carbon metabolism
Os02g0827400	LOC_Os02g58100		1.5	20.3	-3.7	Similar to predicted protein	Carbon metabolism
Os01g0700100	LOC_Os01g50460	*OsSWEET2b*	3.8	38.0	-3.3	MtN3 and saliva related transmembrane protein family protein	Carbon metabolism
Os04g0604300	LOC_Os04g51460	*OsXTH1*	0.1	5.1	-5.6	Glycosyl hydrolases family 16, putative, expressed	Cell wall degradation
Os03g0678800	LOC_Os03g47530		0.8	14.8	-4.2	Glycosyl transferase, family 8 protein	Cell wall degradation
Os03g0301200	LOC_Os03g18910	*OsPCS13*	0.8	13.5	-4.1	Glycosyl-phosphatidyl inositol-anchored, COBRA-like protein 7 precursor, putative, expressed	Cell wall degradation
Os04g0571600	LOC_Os04g48290		3.7	61.4	-4.1	Multi antimicrobial extrusion protein, MatE family protein	Detoxification
Os01g0905200	LOC_Os01g67810	*OsEXO70FX15*	0.4	10.5	-4.6	Exo70 exocyst complex subunit family protein, transposon protein	Exocytosis
Os01g0905300	LOC_Os01g67820	*OsEXO70FX14*	0.1	1.1	-4.2	Exo70 exocyst complex subunit domain containing protein	Exocytosis
Os03g0192700	LOC_Os03g09250	*OsRINO1, MIPS*	0.4	9.6	-4.6	Similar to Myo-inositol-1-phosphate synthase, inositol-3-phosphate synthase	Lipid synthesis

Supplementary Table S3. Continued.

Gene ID	MSU ID	Gene name	T8	C8	Log_2	Description	Classification
Os02g0770800	LOC_Os02g53130	*OsNIA1*	0.1	2.3	-4.9	Similar to Nitrate reductase [NAD(P)H]	Nitrogen remobilisation
Os04g0509600	LOC_Os04g43070	*OsAMT1*	15.7	155.5	-3.3	Similar to Ammonium transporter 1 member 1	Nitrogen remobilisation
Os02g0176000	LOC_Os02g07930	*OsBBX2*	8.3	97.9	-3.6	Zinc finger, B-box domain containing protein	Nucleic acid metabolism
Os02g0815700	LOC_Os02g57060	*OsCttP2*	0.1	3.6	-5.1	Similar to D1 protease (Fragment), Putative C-terminal processing peptidase homologue	Photosynthesis
Os08g0137800	LOC_Os08g04340	*OsUCL24*	1.2	13.8	-3.5	plastocyanin-like domain containing protein, putative, expressed, Cupredoxin domain containing protein	Photosynthesis
Os11g0151400	LOC_Os11g05380		0.3	34.1	-6.8	Cytochrome P450 family protein	Phytohormone related
Os01g0768333	LOC_Os01g56240	*OsSAUR2*	0.3	10.4	-5.0	Auxin responsive SAUR protein family protein	Phytohormone related
Os12g0150200	LOC_Os12g05440	*OsCYP450*	0.1	2.2	-4.5	Cytochrome P450 enzyme	Phytohormone related
Os09g0546900	LOC_Os09g37480	*OsSAUR53*	0.3	7.8	-4.5	Similar to Auxin induced protein	Phytohormone related
Os09g0461500	LOC_Os09g28690		0.5	9.6	-4.2	Alpha/beta hydrolase fold-3 domain containing protein, gibberellin receptor GID1L2	Phytohormone related
Os02g0703600	LOC_Os02g47470	*OsCYP707A5*	0.6	11.4	-4.2	Cytochrome P450 enzyme	Phytohormone related
Os04g0526800	LOC_Os04g44500		0.5	7.4	-3.9	GRAM domain containing protein, GEM, putative, expressed	Phytohormone related
Os01g0699600	LOC_Os01g50420		1.1	44.1	-5.4	STE_MEKK_ste11_MAP3K.7 - STE kinases include homologs to sterile 7, sterile 11 and sterile 20 from yeast, expressed	Plant development process
Os01g0642000	LOC_Os01g45460		0.1	2.1	-4.0	Carboxylesterase, type B family protein	Plant development process
Os10g0330400	LOC_Os10g18370		2.2	33.3	-3.9	Transcriptional regulator, putative, expressed	Plant development process
Os01g0699500	LOC_Os01g50410	*OsMAP3K6*	1.0	24.9	-4.6	Serine/threonine protein kinase domain containing protein	Protein phosphorylation
Os01g0892800	LOC_Os01g66860	*OsSTA40*	0.9	10.7	-3.5	Serine/threonine protein kinase domain containing protein	Protein phosphorylation
Os02g0548700	LOC_Os02g34410	*OsPUB43*	0.0	1.0	-4.6	Similar to ubiquitin-protein ligase, U-box domain-containing protein	Protein ubiquitination

Supplementary Table S3. Continued.

Gene ID	MSU ID	Gene name	T8	C8	Log_2	Description	Classification
Os02g0682300	LOC_Os02g45780		1.2	28.4	-4.5	Zinc finger, C3HC4 type domain containing protein	Protein ubiquitination
Os02g0540700	LOC_Os02g33680	*OsPUB45*	4.2	73.9	-4.2	U-box domain containing protein, expressed	Protein ubiquitination
Os02g0539200	LOC_Os02g33590	*OsPUB33*	0.3	5.0	-4.0	Zinc finger, RING/FYVE/PHD-type domain containing protein	Protein ubiquitination
Os12g0117600	LOC_Os12g02540		0.1	0.7	-3.8	Broad Complex BTB domain with non-phototropic hypocotyl 3 NPH3 and coiled-coil domains, expressed	Protein ubiquitination
Os04g0385600	LOC_Os04g31610	*OsFBO3*	0.5	7.0	-3.8	Tetratricopeptide-like helical domain containing protein	Protein ubiquitination
Os08g0187900	LOC_Os08g08850		7.1	97.1	-3.8	Ubiquitin-conjugating enzyme, E2 domain containing protein	Protein ubiquitination
Os01g0972000	LOC_Os01g74040	*OsRFPH2-3*	4.8	50.3	-3.4	Zinc finger, RING/FYVE/PHD-type domain containing protein	Protein ubiquitination
Os04g0301500	LOC_Os04g23550	*OsbHLH006, RERJ1*	0.3	23.1	-6.2	Helix-loop-helix DNA-binding domain containing protein	TF-bHLH Familiy; Jasmonate signalling
Os03g0741100	LOC_Os03g53020	*OsbHLH148*	5.4	65.6	-3.6	Basic helix-loop-helix transcription factor, Drought tolerance	TF-bHLH Family; Protein synthesis
Os03g0820400	LOC_Os03g60570	*OsZFP15*	0.8	24.3	-5.0	C2H2 zinc finger protein, expressed	TF-C2H2 Family; plant growth and stress
Os01g0264000	LOC_Os01g15900	*OsDof, OsDof2*	0.7	9.6	-3.7	Zinc finger, Dof-type domain containing protein	TF-Dof Family; Protein synthesis
Os08g0474000	LOC_Os08g36920	*OsERF104*	0.9	41.5	-5.6	AP2 / ERF domain containing protein, expressed	TF-ERF Family; Ethylene response factor
Os02g0677300	LOC_Os02g45450	*OsERF025*	1.5	43.4	-4.8	Dehydration-responsive element-binding protein, putative, expressed	TF-ERF Family; Ethylene response factor
Os02g0676800	LOC_Os02g45420	*OsERF20, OsEFR126*	0.0	0.9	-4.2	AP2 / ERF domain containing protein, expressed	TF-ERF Family; Ethylene response factor
Os10g0562900	LOC_Os10g41330	*OsERF96, OsDERF12*	9.8	140.5	-3.8	AP2 / ERF domain containing protein, expressed	TF-ERF Family; Ethylene response factor

Supplementary Table S3. Continued.

Gene ID	MSU ID	Gene name	T8	C8	Log_2	Description	Classification
Os09g0522000	LOC_Os09g35010	*OsERF31*	20.3	280.3	-3.8	Dehydration-responsive element-binding protein, putative, expressed	TF-ERF Family; Ethylene response factor
Os09g0522200	LOC_Os09g35030	*OsERF24, OsDREB1A*	4.7	63.8	-3.8	Dehydration-responsive element-binding protein, putative, expressed	TF-ERF Family; Ethylene response factor
Os03g0191900	LOC_Os03g09170	*OsERF47*	0.9	11.9	-3.7	AP2 / ERF domain containing protein, expressed	TF-ERF Family; Ethylene response factor
Os01g0885900	LOC_Os01g66270	*OsERF17*	0.3	3.5	-3.3	AP2 / ERF domain containing protein, expressed	TF-ERF Family; Ethylene response factor
Os02g0649300	LOC_Os02g43330	*OsHOX24*	0.1	3.9	-5.0	homeobox associated leucine zipper, putative, expressed	TF-HD-ZIP Family; plant growth and stress response
Os02g0232000	LOC_Os02g13800	*OsHsfC2a*	0.6	5.9	-3.4	HSF-type DNA-binding domain containing protein, expressed	TF-HSF Family; Protein synthesis
Os01g0511000	LOC_Os01g32770		0.7	9.4	-3.7	LOB domain-containing protein 40	TF-LBD Family; plant growth and stress response
Os04g0517100	LOC_Os04g43680	*OsMYB4*	1.6	19.3	-3.6	MYB family transcription factor, putative, expressed, Similar to OSIGBa0145M07.4 protein	TF-MYB Family; plant growth and stress response
Os02g0685200	LOC_Os02g46030	*OsMyb1R*	0.5	7.6	-4.0	MYB family transcription factor, putative, expressed	TF-MYB_related Family; plant growth and stress response
Os08g0157600	LOC_Os08g06110	*OsCCA1, OsLHY*	37.9	493.1	-3.7	MYB family transcription factor, putative, expressed	TF-MYB_related Family; plant growth and stress response
Os12g0123700	LOC_Os12g03040	*ONAC131*	0.4	4.9	-3.8	No apical meristem protein, putative, expressed	TF-NAC Family; plant growth and stress response
Os03g0815100	LOC_Os03g60080	*OsNAC9*	31.6	350.5	-3.5	NAC domain-containing protein 67, putative, expressed	TF-NAC Family; plant growth and stress response
Os01g0675800	LOC_Os01g48446	*ONAC14*	2.9	30.5	-3.4	no apical meristem protein, putative, expressed	TF-NAC Family; plant growth and stress response
Os10g0391400	LOC_Os10g25230	*OsJAZ13, OsTIFY11e*	0.5	23.3	-5.4	Tify domain containing protein, ZIM domain containing protein	TF-Tify family; Jasmonate signalling

Supplementary Table S3. Continued.

Gene ID	MSU ID	Gene name	T8	C8	Log_2	Description	Classification
Os03g0181100	LOC_Os03g08330	*OsJAZ 10, OsTIFY11b*	6.2	155.1	-4.6	Tify domain containing protein, ZIM domain containing protein	TF-Tify family; Jasmonate signalling
Os10g0392400	LOC_Os10g25290	*OsJAZ1, OsTIFY11d*	1.7	27.4	-4.0	Tify domain containing protein, ZIM domain containing protein	TF-Tify family; Jasmonate signalling
Os03g0180800	LOC_Os03g08310	*OsJAZ9, OsTIFY11a*	0.3	4.5	-4.0	Tify domain containing protein, ZIM domain containing protein	TF-Tify family; Jasmonate signalling
Os01g0821600	LOC_Os01g60640	*OsWRKY21*	2.3	34.3	-3.9	WRKY transcription factor 21	TF-WRKY Family; Protein synthesis
Os02g0181300	LOC_Os02g08440	*OsWRKY71*	55.1	593.6	-3.4	WRKY transcription factor 71	TF-WRKY Family; Protein synthesis
Os11g0118500	LOC_Os11g02620		0.1	2.1	-3.9	Similar to protein binding / signal transducer	Ungrouped
Os12g0189300	LOC_Os12g08760		17.7	251.3	-3.8	carboxyvinyl-carboxyphosphonate phosphorylmutase, putative, expressed, Pyruvate/Phosphoenolpyruvate kinase, catalytic core domain containing protein	Ungrouped
Os01g0186900	LOC_Os01g09220		7.5	106.1	-3.8	transposon protein, putative, CACTA, En/Spm sub-class, expressed	Ungrouped
Os01g0975000	LOC_Os01g74370	*OsDSR3*	0.1	1.2	-3.7	Protein of unknown function DUF966 family protein	Ungrouped
Os01g0498300	LOC_Os01g31370		0.2	2.0	-3.7	Glycosyltransferase AER61, uncharacterized domain containing protein	Ungrouped
Os01g0796000	LOC_Os01g58350		3.4	42.2	-3.6	Conserved hypothetical protein	Ungrouped
Os10g0521900	LOC_Os10g37760	*OsRhmbd17*	0.9	11.3	-3.6	Putative Rhomboid homologue, expressed	Ungrouped
Os01g0955100	LOC_Os01g72530	*OsCML31, OsMSR2*	10.2	119.8	-3.6	Similar to Calmodulin-like protein (Fragment)	Ungrouped
Os07g0680600	LOC_Os07g48280		7.7	89.3	-3.5	Protein of unknown function DUF3133 domain containing protein	Ungrouped
Os12g0510750	LOC_Os12g32610		3.7	81.5	-4.4	Conserved hypothetical protein	Unknown
Os01g0952900	LOC_Os01g72360		0.4	7.0	-4.3	Conserved hypothetical protein	Unknown
Os01g0846400	LOC_Os01g62770		0.1	1.6	-4.0	Conserved hypothetical protein	Unknown

Supplementary Table S3. Continued.

Gene ID	MSU ID	Gene name	T8	C8	Log_2	Description	Classification
Os06g0683800	LOC_Os06g46980		0.1	1.4	-3.9	Conserved hypothetical protein	Unknown
Os05g0181700	LOC_Os05g08900		3.3	49.8	-3.9	Conserved hypothetical protein	Unknown
Os05g0552800	LOC_Os05g47960		5.0	74.6	-3.9	Conserved hypothetical protein	Unknown
Os06g0133500	LOC_Os06g04240		2.9	41.3	-3.8	Conserved hypothetical protein	Unknown
Os01g0263900			2.6	32.5	-3.7	Hypothetical protein	Unknown
Os01g0195300	LOC_Os01g09870		0.2	2.7	-3.5	Hypothetical conserved gene	Unknown
Os10g0205700	LOC_Os10g13850		2.3	26.5	-3.5	Pollen Ole e 1 allergen/extensin domain containing protein	Unknown
Os05g0519300	LOC_Os05g44300		2.4	26.7	-3.5	Plant-specific domain TIGR01615 family protein, expressed	Unknown
Os01g0795600	LOC_Os01g58310		2.2	23.3	-3.4	Conserved hypothetical protein	Unknown
Os03g0734500	LOC_Os03g52410		7.3	78.4	-3.4	Conserved hypothetical protein	Unknown
Os02g0733900	LOC_Os02g50110		0.4	4.2	-3.4	Conserved hypothetical protein	Unknown
Os01g0389200	LOC_Os01g29280		0.3	2.8	-3.4	Expressed protein	Unknown
Os02g0758200	LOC_Os02g52170		1.8	18.6	-3.3	Conserved hypothetical protein	Unknown
Os05g0148800	LOC_Os05g05610		0.1	1.1	-3.3	Hypothetical conserved gene	Unknown
