# Supplementary material for: Transcriptional response of rice flag leaves to restricted external phosphorus supply during grain filling in rice cv. IR64
Source: PLoS One. 2018 Sep 13;13(9):e0203654. doi: 10.1371/journal.pone.0203654 (PMC6136725; doi:10.1371/journal.pone.0203654)
Supplement: S4 Table — (PDF) [file pone.0203654.s007.pdf]

Supplementary Table S4. The list of 100 of most each up and downregulated differential expressed genes in T16.

Gene ID	MSU ID	Gene name	T16	C16	Log_2	Description	Classification
**100 UP regulated in T16**							
Os02g0712700	LOC_Os02g48210		2.0	0.2	3.1	Concanavalin A-like lectin/glucanase domain containing protein	Abiotic / biotic stress response
Os05g0355450			1056.5	120.8	3.1	Hypothetical protein	Abiotic / biotic stress response
Os05g0355400	LOC_Os05g28740	universal stress-induced protein A	1252.7	143.9	3.1	Universal stress protein domain containing protein, putative, expressed	Abiotic / biotic stress response
Os12g0552400	LOC_Os12g36630		2.6	0.4	2.9	Universal stress protein domain containing protein, putative, expressed	Abiotic / biotic stress response
Os03g0184550	LOC_Os03g08624		5.0	0.8	2.6	dihydroflavonol-4-reductase, putative, expressed	Abiotic / biotic stress response
Os02g0731500	LOC_Os02g49860		1.7	0.3	2.3	AWPM-19-like membrane family protein, putative, expressed	Abiotic / biotic stress response
Os05g0125300	LOC_Os05g03460	*OsRLCK177*	1.1	0.2	2.3	Protein kinase, putative, expressed	Abiotic / biotic stress response
Os04g0344100	LOC_Os04g27670		1.4	0.3	2.3	Terpene synthase family, metal binding domain containing protein, expressed	Abiotic / biotic stress response
Os10g0565200	LOC_Os10g41550		21.5	1.5	3.9	beta-amylase, putative, expressed	Carbohydrate metabolism
Os09g0255300	LOC_Os09g08120		4.3	0.8	2.3	beta-fructofuranosidase, insoluble isoenzyme 7 precursor, putative, expressed	Carbohydrate metabolism
Os06g0335900	LOC_Os06g22919	*OsXTH15*	7.2	0.9	3.0	Concanavalin A-like lectin/glucanase domain containing protein.	Cell wall degradation
Os06g0335950	LOC_Os06g22919		2.3	0.3	2.9	DEFL9 - Defensin and Defensin-like DEFL family, expressed	Cell wall degradation
Os05g0375400	LOC_Os05g31140		128.2	19.7	2.7	glycosyl hydrolases family 17, putative, expressed	Cell wall degradation
Os08g0495800	LOC_Os08g38710		7.1	0.5	3.9	uncharacterized glycosyltransferase, putative, expressed	Cell wall degradation

Supplementary Table S4. Continued.

Gene ID	MSU ID	Gene name	T16	C16	Log_2	Description	Classification
Os04g0630600	LOC_Os04g53830		1.3	0.3	2.3	3-beta hydroxysteroid dehydrogenase/isomerase family protein, putative, expressed	Detoxification
Os07g0684100	LOC_Os07g48510		28.3	3.1	3.2	Thioredoxin, putative, expressed	Detoxification
Os05g0171900	LOC_Os05g07940		10.5	1.3	3.0	glyoxalase family protein, putative, expressed	Detoxification
Os11g0655900	LOC_Os11g43520	*OsGRX23*	14.4	1.9	3.0	glutaredoxin subgroup III, expressed; Thioredoxin fold domain containing protein	Detoxification
Os06g0306300	LOC_Os06g20150		3.0	0.5	2.6	peroxidase precursor, putative, expressed	Detoxification
Os02g0240300	LOC_Os02g14440		5.1	0.9	2.5	peroxidase precursor, putative, expressed	Detoxification
Os03g0120400	LOC_Os03g02860		9.8	1.8	2.5	heavy metal-associated domain containing protein, expressed	Detoxification
Os09g0498400	LOC_Os09g32290		69.2	13.8	2.3	FAD dependent oxidoreductase domain containing protein, expressed	Detoxification
Os07g0529000	LOC_Os07g34520	*OsI85*	1.2	0.1	3.1	isocitrate lyase, putative, expressed	Glycolysis
Os04g0608100	LOC_Os04g51880		21.5	3.6	2.6	GHMP kinases ATP-binding protein, putative, expressed	Glycolysis
Os01g0871900	LOC_Os01g65140		7.7	0.7	3.5	Peptide transporter PTR2, putative, expressed, Similar to POT family protein	Nitrogen remobilisation
Os01g0871800	LOC_Os01g65130		1.2	0.1	3.5	peptide transporter, putative, expressed	Nitrogen remobilisation
Os05g0231700	LOC_Os05g14240	*OsTIP4;1*	309.3	39.9	3.0	aquaporin protein, putative, expressed	Nitrogen remobilisation
Os01g0872000	LOC_Os01g65150		4.9	0.7	2.8	TGF-beta receptor, type I/II extracellular region family protein	Nitrogen remobilisation
Os02g0580900	LOC_Os02g37040		16.0	2.7	2.5	peptide transporter PTR3-A, putative, expressed	Nitrogen remobilisation
Os07g0100600	LOC_Os07g01070		1.5	0.3	2.3	peptide transporter, putative, expressed	Nitrogen remobilisation
Os01g0102850			23.1	1.8	3.7	Similar to nitrilase 2	Nitrogen remobilisation
Os04g0679400	LOC_Os04g58280		156.0	26.2	2.6	stem-specific protein TSJT1, putative, expressed	Nitrogen remobilisation
Os08g0113700	LOC_Os08g02180		8.8	1.5	2.5	expressed protein	Nitrogen remobilisation

Supplementary Table S4. Continued.

Gene ID	MSU ID	Gene name	T16	C16	Log_2	Description	Classification
Os05g0555600	LOC_Os05g48200		118.3	20.8	2.5	Glutamate synthase, chloroplast precursor, putative, expressed	Nitrogen remobilisation
Os03g0765400	LOC_Os03g55660, LOC_Os03g55670		14.5	2.2	2.7	nucleoporin, putative, expressed	Nucleic acid metabolism
Os03g0582000	LOC_Os03g38540		86.4	17.0	2.3	Folic acid binding protein, putative, expressed	Nucleic acid metabolism
Os03g0701200	LOC_Os03g49440		2.2	0.4	2.4	Phosphatase, putative, expressed	Phosphatases
Os07g0147550	LOC_Os07g05365		3.8	0.6	2.7	Photosystem II 10 kDa polypeptide, chloroplast precursor, putative, expressed	Photosynthesis
Os11g0671000	LOC_Os11g44810		1084.1	203.6	2.4	auxin-repressed protein, putative, expressed	Phytohormone related
Os09g0447300	LOC_Os09g27500		2.8	0.5	2.3	Cytochrome P450, putative, expressed	Phytohormone related
Os02g0571300	LOC_Os02g36220	*OsKS5*	2.5	0.5	2.3	Ent-pimara-8(14),15-diene synthase, terpene synthase, putative, expressed	Phytohormone related
Os03g0275300	LOC_Os03g16780		37.6	5.1	2.9	Ankyrin repeat family protein, putative, expressed	Protein modification
Os10g0180800	LOC_Os10g10130	*OsWAK112d*	270.0	55.6	2.3	OsWAK receptor-like protein kinase, expressed	Protein phosphorylation
Os05g0453500	LOC_Os05g37950		8.2	1.5	2.4	Ganylyl cyclase, putative, expressed	Protein synthesis
Os12g0117600	LOC_Os12g02540		1.7	0.1	4.9	Broad Complex BTB domain with non-phototropic hypocotyl 3 NPH3 and coiled-coil domains, expressed	Protein ubiquitination
Os02g0159800	LOC_Os02g06470	*OsFBX38*	3.5	0.2	4.4	Cyclin-like F-box domain containing protein,	Protein ubiquitination
Os10g0183800	LOC_Os10g10420	*OsFBX378*	7.3	0.5	3.9	Similar to F-box domain containing protein	Protein ubiquitination
Os05g0478000	LOC_Os05g40020		17.2	1.4	3.7	RING-H2 finger protein, putative, expressed	Protein ubiquitination
Os03g0435300	LOC_Os03g32100		4.9	0.6	3.1	Spotted leaf 11, putative, expressed, Similar to exodeoxyribonuclease V	Protein ubiquitination
Os07g0593000	LOC_Os07g40300		2.6	0.3	2.9	ZOS7-10 - C2H2 zinc finger protein, expressed, Similar to Zinc-finger protein	Protein ubiquitination
Os05g0172000	LOC_Os05g07950	*OsFBX160*	23.9	3.2	2.9	F-box domain containing protein	Protein ubiquitination
Os05g0360400	LOC_Os05g29710		21.1	3.4	2.6	RING-H2 finger protein, putative, expressed	Protein ubiquitination

Supplementary Table S4. Continued.

Gene ID	MSU ID	Gene name	T16	C16	Log_2	Description	Classification
Os06g0534900	LOC_Os06g34400		19.6	3.2	2.6	zinc finger, C3HC4 type domain containing protein, expressed	Protein ubiquitination
Os09g0554200	LOC_Os09g38110		2.8	0.6	2.3	RING-H2 finger protein, putative, expressed	Protein ubiquitination
Os03g0826900	LOC_Os03g61160	*OsEnS-58*	12.4	1.8	2.8	Conserved hypothetical protein	Seed storage
Os12g0428000	LOC_Os12g24020	Sulfurtransferase 7, *OsStr7*	1.5	0.1	3.6	Similar to senescence-associated protein DIN1	Senescence
Os02g0661900	LOC_Os02g44300		7.0	0.7	3.3	Similar to Vesicle-associated membrane protein-associated protein B/C (VAMP-associated protein B/C)	Senescence
Os05g0525900	LOC_Os05g45020	*OsC3H37*	3.6	0.5	2.7	zinc finger/CCCH transcription factor, putative, expressed	TF-C3H family; Protein synthesis
Os06g0264200	LOC_Os06g15330	*OsCCT20*	56.4	10.5	2.4	CCT/B-box zinc finger protein, putative, expressed	TF-CO-like Family; Protein synthesis
Os12g0209200	LOC_Os12g10660	*OsBBX30*	2.5	0.4	2.5	B-box zinc finger family protein, putative, expressed	TF-DBB Family; Protein ubiquitination
Os01g0971800	LOC_Os01g74020	*OsPCL1*	21.6	1.8	3.6	MYB family transcription factor, putative, expressed	TF-G2-like Family; plant growth and stress response
Os03g0766500	LOC_Os03g55760	*OsKANADI4*	5.5	1.0	2.4	MYB family transcription factor, putative, expressed	TF-G2-like Family; Protein synthesis
Os09g0538400	LOC_Os09g36730	*OsMYB108*	19.2	2.7	2.8	MYB family transcription factor, putative, expressed	TF-MYB Family; Protein synthesis
Os02g0187700	LOC_Os02g09480		6.2	1.3	2.3	myb-like DNA-binding domain containing protein, putative, expressed	TF-MYB Family; Protein synthesis
Os04g0583900	LOC_Os04g49450	*OsMYB511*	1.2	0.2	3.0	MYB family transcription factor, putative, expressed	TF-MYB_related Family; plant growth and stress response
Os02g0685200	LOC_Os02g46030	*OsMyb1R*	2.2	0.4	2.3	MYB family transcription factor, putative, expressed	TF-MYB_related Family; plant growth and stress response
Os10g0578600	LOC_Os10g42770		2.8	0.2	3.8	Methyltransferase type 11 domain containing protein	Ungrouped

Supplementary Table S4. Continued.

Gene ID	MSU ID	Gene name	T16	C16	Log_2	Description	Classification
Os11g0118500	LOC_Os11g02620		2.7	0.2	3.5	Similar to protein binding / signal transducer	Ungrouped
Os01g0930950	LOC_Os01g70540		80.1	11.5	2.8	NAD(P)-binding domain domain containing protein	Ungrouped
Os01g0237000	LOC_Os01g13560		5.9	0.9	2.6	membrane associated DUF588 domain containing protein, putative, expressed	Ungrouped
Os03g0184100	LOC_Os03g08580		7.8	0.3	4.9	Conserved hypothetical protein	Unknown
Os11g0118400			5.5	0.3	4.4	Hypothetical protein	Unknown
Os08g0495866			17.7	0.9	4.2	Hypothetical protein	Unknown
Os10g0565150			42.2	2.3	4.2	Hypothetical gene	Unknown
Os02g0674233	LOC_Os02g45225		5.9	0.4	3.8	Hypothetical conserved gene	Unknown
Os03g0826800	LOC_Os03g61150		94.4	8.1	3.5	Conserved hypothetical protein	Unknown
Os02g0674400			1.1	0.1	3.5	Conserved hypothetical protein	Unknown
Os07g0618700	LOC_Os07g42650		2.3	0.3	3.2	Conserved hypothetical protein	Unknown
Os06g0474800	LOC_Os06g28050		35.9	4.4	3.0	Similar to 3Fe-4S ferredoxin	Unknown
Os10g0181100	LOC_Os10g10149		5.3	0.7	3.0	Conserved hypothetical protein	Unknown
Os05g0142400	LOC_Os05g05060		92.2	12.1	2.9	Conserved hypothetical protein	Unknown
Os04g0583200	LOC_Os04g49370		9.8	1.4	2.8	Conserved hypothetical protein	Unknown
Os01g0923000	LOC_Os01g69870		58.3	8.5	2.8	Conserved hypothetical protein	Unknown
Os01g0101850			3.0	0.4	2.8	Hypothetical protein	Unknown
Os01g0665900	LOC_Os01g47570		4.9	0.7	2.8	Conserved hypothetical protein	Unknown
Os06g0267450			3.3	0.5	2.7	Hypothetical protein	Unknown
Os02g0756100	LOC_Os02g51950		3.1	0.5	2.7	Conserved hypothetical protein	Unknown
Os03g0272650			14.6	2.2	2.7	Non-protein coding transcript	Unknown
Os04g0684100	LOC_Os04g58734		3.9	0.6	2.7	expressed protein	Unknown
Os01g0872201			21.6	3.4	2.7	Hypothetical protein	Unknown

Supplementary Table S4. Continued.

Gene ID	MSU ID	Gene name	T16	C16	Log_2	Description	Classification
Os09g0443400	LOC_Os09g27135		13.7	2.3	2.6	expressed protein	Unknown
Os04g0583266			15.3	2.6	2.5	Hypothetical protein	Unknown
Os11g0671100			189.8	32.6	2.5	Hypothetical protein	Unknown
Os06g0263801			19.0	3.4	2.5	Hypothetical gene	Unknown
Os04g0679500			72.8	13.0	2.5	Hypothetical protein	Unknown
Os03g0120425			5.3	1.0	2.5	Non-protein coding transcript	Unknown
Os02g0525501	LOC_Os02g32465		10.3	1.9	2.5	expressed protein	Unknown
Os09g0128400	LOC_Os09g04160		59.5	12.1	2.3	expressed protein	Unknown
Os02g0580966			23.8	4.9	2.3	Hypothetical protein	Unknown
Os12g0211900	LOC_Os12g10880		2.2	0.5	2.3	expressed protein	Unknown
**100 DOWN regulated in T16**							
Os05g0582000	LOC_Os05g50500		0.8	16.3	-4.3	secretory protein, putative, expressed	Abiotic / biotic stress response
Os06g0694200	LOC_Os06g47910	*OsGELP88*	0.1	1.4	-4.0	GDSL-like lipase/acylhydrolase, putative, expressed	Abiotic / biotic stress response
Os08g0520600	LOC_Os08g40910		0.2	2.9	-3.7	Conserved hypothetical protein	Abiotic / biotic stress response
Os09g0444900	LOC_Os09g27260		0.2	1.5	-3.1	plant viral response family protein, putative, expressed	Abiotic / biotic stress response
Os11g0215100	LOC_Os11g10870		1.0	6.9	-2.8	dirigent, putative, expressed	Abiotic / biotic stress response
Os02g0258800	LOC_Os02g15860		2.9	18.4	-2.7	expressed protein	Abiotic / biotic stress response
Os12g0199000	LOC_Os12g09720		6.4	40.7	-2.7	dirigent, putative, expressed	Abiotic / biotic stress response
Os02g0823100	LOC_Os02g57720	*OsPIP1*;3	7.9	49.4	-2.6	aquaporin protein, putative, expressed	Abiotic / biotic stress response

Supplementary Table S4. Continued.

Gene ID	MSU ID	Gene name	T16	C16	Log_2	Description	Classification
Os10g0533500	LOC_Os10g38940	*OsHYD2*	0.3	1.7	-2.6	fatty acid hydroxylase, putative, expressed	Abiotic / biotic stress response
Os04g0684900	LOC_Os04g58810	*OsCAF1B*	1.8	10.0	-2.5	CAF1 family ribonuclease containing protein, putative, expressed	Abiotic / biotic stress response
Os07g0523400	LOC_Os07g33910	*OsGPT2-3*	0.1	1.0	-2.8	transporter family protein, putative, expressed	Carbon metabolism
Os12g0476200	LOC_Os12g29220	*OsSWEET13*	0.3	2.0	-2.5	nodulin MtN3 family protein, putative, expressed	Carbon metabolism
Os03g0301200	LOC_Os03g18910		0.1	1.0	-3.0	Glycosyl-phosphatidyl inositol-anchored, plant domain containing protein	Cell wall degradation
Os01g0194600	LOC_Os01g09830	*OsGRX2*	0.3	2.5	-2.9	glutaredoxin subgroup III, expressed	Detoxification
Os07g0694700	LOC_Os07g49400	*OsAPx2*	12.1	83.8	-2.8	Cytosolic Ascorbate Peroxidase encoding gene 4,5,6,8, expressed	Detoxification
Os01g0847700	LOC_Os01g62870	*OsAKR2*	0.5	3.2	-2.6	oxidoreductase, aldo/keto reductase family protein, putative, expressed	Detoxification
Os10g0532300	LOC_Os10g38870		3.8	22.4	-2.6	heavy metal-associated domain containing protein, expressed	Detoxification
Os02g0814000	LOC_Os02g56900		2.2	12.8	-2.5	thioredoxin family protein, putative, expressed	Detoxification
Os04g0561500	LOC_Os04g47360	*OsPOP9*	9.1	88.5	-3.3	Putative Prolyl Oligopeptidase homologue, expressed	Nitrogen remobilisation
Os01g0975900	LOC_Os01g74450	*OsTIP1;2*	27.9	208.5	-2.9	aquaporin protein, putative, expressed	Nitrogen remobilisation
Os02g0116300	LOC_Os02g02450	*OsYs4, OsYSL7*	0.2	1.4	-2.8	Oligopeptide transporter OPT superfamily protein / Similar to Yellow stripe-like transporter 17, putative, unclassified, expressed	Nitrogen remobilisation
Os03g0701100	LOC_Os03g49430		1.9	15.2	-3.0	pre-mRNA-splicing factor, putative, expressed	Nucleic acid metabolism
Os05g0551700	LOC_Os05g47840		0.3	1.8	-2.6	IPP transferase, putative, expressed	Nucleic acid metabolism
Os07g0178700	LOC_Os07g08150		0.2	19.2	-6.9	early light-induced protein, chloroplast precursor, putative, expressed	Photosynthesis
Os07g0178800	LOC_Os07g08160		1.2	107.7	-6.5	early light-induced protein, chloroplast precursor, putative, expressed	Photosynthesis

Supplementary Table S4. Continued.

Gene ID	MSU ID	Gene name	T16	C16	Log_2	Description	Classification
Os01g0246400	LOC_Os01g14410		6.7	72.1	-3.4	early light-induced protein, chloroplast precursor, putative, expressed	Photosynthesis
Os04g0690800	LOC_Os04g59440	*PsbS, OsPsbS2, PSII-S*	4.8	37.3	-3.0	chlorophyll A-B binding protein, putative, expressed	Photosynthesis
Os01g0186900	LOC_Os01g09220		0.8	8.4	-3.4	transposon protein, putative, CACTA, En/Spm sub-class, expressed.	Phytohormone related
Os08g0479300	LOC_Os08g37390		0.1	0.9	-2.9	cyclin, putative, expressed	Plant development process
Os01g0699600	LOC_Os01g50420		0.3	2.1	-2.8	Serine/threonine protein kinase domain containing protein	Protein phosphorylation
Os06g0253100	LOC_Os06g14240		0.4	28.4	-6.1	hsp20/alpha crystallin family protein, putative, expressed	Protein synthesis
Os02g0758000	LOC_Os02g52150	*OsHsp24.1*	1.6	37.6	-4.6	heat shock 22 kDa protein, mitochondrial precursor, putative, expressed	Protein synthesis
Os05g0562300	LOC_Os05g48810	*OsDjB7*	0.6	5.4	-3.2	dnaJ domain containing protein, expressed	Protein synthesis
Os02g0782500	LOC_Os02g54140		0.4	3.5	-3.0	hsp20/alpha crystallin family protein, putative, expressed	Protein synthesis
Os03g0267000	LOC_Os03g16030		0.3	2.4	-2.8	hsp20/alpha crystallin family protein, putative, expressed	Protein synthesis
Os02g0782300	LOC_Os02g54130		0.8	4.9	-2.7	heat shock protein DnaJ, putative, expressed	Protein synthesis
Os03g0266900	LOC_Os03g16020		0.5	3.2	-2.6	hsp20/alpha crystallin family protein, putative	Protein synthesis
Os06g0195800	LOC_Os06g09560		0.4	2.5	-2.6	heat shock protein DnaJ, putative, expressed	Protein synthesis
Os02g0139100	LOC_Os02g04650		2.1	11.8	-2.5	activator of 90 kDa heat shock protein ATPase homolog, putative, expressed	Protein synthesis
Os02g0759900	LOC_Os02g52260		9.9	93.1	-3.2	expressed protein	Protein systhesis
Os10g0154700	LOC_Os10g06630		2.1	11.8	-2.5	peptidyl-prolyl cis-trans isomerase, putative, expressed	Protein systhesis
Os02g0540700	LOC_Os02g33680		0.3	4.3	-3.8	U-box domain containing protein, expressed	Protein ubiquitination
Os02g0759400	LOC_Os02g52210	*OsRING-1*	1.0	10.8	-3.5	zinc finger, C3HC4 type domain containing protein, expressed	Protein ubiquitination

Supplementary Table S4. Continued.

Gene ID	MSU ID	Gene name	T16	C16	Log_2	Description	Classification
Os08g0415600	LOC_Os08g32060	*OsPUB5*	0.1	1.2	-3.3	spotted leaf 11, putative, expressed	Protein ubiquitination
Os02g0686100	LOC_Os02g46100		0.3	2.0	-2.9	RING-H2 finger protein, putative, expressed	Protein ubiquitination
Os01g0555100	LOC_Os01g37460	*SRZ3*	0.4	2.9	-2.7	zinc finger family protein, putative, expressed	Protein ubiquitination
Os02g0779000	LOC_Os02g53850	*OsSub21*	2.0	12.5	-2.7	Putative Subtilisin homologue, expressed	Protein ubiquitination
Os03g0427300	LOC_Os03g31360	*GLUA3*	0.2	11.4	-6.1	glutelin, putative, expressed; Seed storage protein	Seed storage
Os01g0762500	LOC_Os01g55690		0.4	17.3	-5.3	glutelin, putative, expressed; Seed storage protein	Seed storage
Os02g0453600	LOC_Os02g25640		0.3	3.5	-3.6	glutelin, putative, expressed	Seed storage
Os06g0198500	LOC_Os06g09820		0.3	2.6	-3.4	cupin superfamily protein, putative, expressed	Seed storage
Os07g0573900	LOC_Os07g38630	*OsAGP27, Arabinogalactan protein 27*	6.5	48.1	-2.9	hypothetical protein	Seed storage
Os06g0124400	LOC_Os06g03390		1.2	6.5	-2.5	expressed protein	Seed storage
Os02g0181900	LOC_Os02g08490		0.9	9.9	-3.4	chaperone protein clpB 1, putative, expressed	Senescence
Os04g0301500	LOC_Os04g23550	*OsbHLH006, RERJ1*	0.1	0.8	-2.5	basic helix-loop-helix family protein, putative, expressed	TF-bHLH Family; Jasmonate signalling
Os09g0431900	LOC_Os09g26210		1.5	8.7	-2.6	C2H2 zinc finger protein, expressed	TF-C2H2 Family; Protein ubiquitination
Os09g0522100	LOC_Os09g35020	*OsERF133*	0.3	3.8	-3.9	AP2 domain containing protein, expressed	TF-ERF Family; Ethylene response
Os08g0474000	LOC_Os08g36920	*OsERF104*	0.1	1.3	-3.7	AP2 domain containing protein, expressed	TF-ERF Family; Ethylene response
Os09g0522000	LOC_Os09g35010	*OsERF31*	2.8	25.0	-3.2	Dehydration-responsive element-binding protein, putative, expressed	TF-ERF Family; Ethylene response
Os04g0610400	LOC_Os04g52090	*OsERF77*	5.0	31.4	-2.7	AP2 domain containing protein, expressed	TF-ERF Family; Ethylene response
Os09g0526600	LOC_Os09g35790	*OsHsfB2c*	0.3	21.4	-6.2	HSF-type DNA-binding domain containing protein, expressed	TF-HSF Family; Protein synthesis

Supplementary Table S4. Continued.

Gene ID	MSU ID	Gene name	T16	C16	Log_2	Description	Classification
Os08g0546800	LOC_Os08g43334	*OsHsfB2b*	0.1	3.9	-5.0	HSF-type DNA-binding domain containing protein, expressed	TF-HSF Family; Protein synthesis
Os04g0568700	LOC_Os04g48030	*OsHsfB2a*	0.5	3.2	-2.7	heat stress transcription factor B-1, putative, expressed	TF-HSF Family; Protein synthesis
Os01g0862800	LOC_Os01g64310	*ONAC59, NAC*	0.2	2.3	-3.4	no apical meristem protein, putative, expressed	TF-NAC Family; plant growth and stress response
Os05g0407100	LOC_Os05g33700		0.8	22.0	-4.7	4F5 protein family protein, expressed	Ungrouped
Os07g0683900	LOC_Os07g48490		35.0	272.0	-3.0	stress responsive protein, putative, expressed	Ungrouped
Os01g0369500	LOC_Os01g27190		0.7	4.3	-2.6	C2 domain containing protein, putative, expressed	Ungrouped
Os09g0526650			0.3	16.4	-5.6	Conserved hypothetical protein	Unknown
Os07g0448150			0.2	4.0	-4.1	Non-protein coding transcript	Unknown
Os04g0521700	LOC_Os04g44100		0.2	2.1	-3.5	expressed protein	Unknown
Os10g0328600			0.8	9.0	-3.5	Hypothetical protein	Unknown
Os12g0199050			9.3	99.2	-3.4	Hypothetical protein	Unknown
Os01g0186950			2.5	26.5	-3.4	Hypothetical protein	Unknown
Os05g0562350			0.7	7.0	-3.3	Hypothetical gene	Unknown
Os03g0734500	LOC_Os03g52410		0.9	8.3	-3.2	Conserved hypothetical protein	Unknown
Os04g0568751			0.7	6.3	-3.1	Hypothetical protein	Unknown
Os02g0198800	LOC_Os02g10530		0.1	1.1	-3.1	expressed protein	Unknown
Os02g0102950			0.2	1.5	-3.0	Hypothetical protein	Unknown
Os01g0276900	LOC_Os01g16980		4.6	35.4	-2.9	Conserved hypothetical protein	Unknown
Os05g0552800	LOC_Os05g47960		0.8	5.7	-2.9	expressed protein	Unknown
Os01g0341300	LOC_Os01g23880		0.4	2.7	-2.8	Conserved hypothetical protein	Unknown
Os02g0507600	LOC_Os02g30420		0.2	1.7	-2.8	Conserved hypothetical protein	Unknown
Os09g0376400	LOC_Os09g20940		0.2	1.1	-2.8	expressed protein	Unknown

Supplementary Table S4. Continued.

Gene ID	MSU ID	Gene name	T16	C16	Log_2	Description	Classification
Os01g0849500	LOC_Os01g63000		1.1	7.4	-2.8	Protein of unknown function DUF3339 domain containing protein	Unknown
Os02g0150250			0.5	3.6	-2.8	Hypothetical gene	Unknown
Os10g0568300	LOC_Os10g41870		0.1	0.9	-2.7	Hypothetical conserved gene	Unknown
Os06g0480500	LOC_Os06g28630		0.7	4.5	-2.7	expressed protein	Unknown
Os09g0412700	LOC_Os09g24620		6.4	42.2	-2.7	expressed protein	Unknown
Os03g0137600	LOC_Os03g04470		1.0	6.3	-2.7	expressed protein	Unknown
Os01g0144800	LOC_Os01g05150		0.4	2.4	-2.6	expressed protein	Unknown
Os02g0303500	LOC_Os02g20040		0.3	1.9	-2.6	loricrin, putative, expressed	Unknown
Os01g0341200	LOC_Os01g23870		0.3	1.7	-2.5	expressed protein	Unknown
Os10g0162100	LOC_Os10g07450		11.0	63.9	-2.5	expressed protein	Unknown
Os05g0124600	LOC_Os05g03390		0.5	3.0	-2.5	expressed protein	Unknown
Os09g0494500	LOC_Os09g32095		0.6	3.5	-2.5	expressed protein	Unknown
Os01g0342500	LOC_Os01g23990		13.8	77.2	-2.5	expressed protein	Unknown
Os11g0594700	LOC_Os11g38210		0.7	3.8	-2.5	Protein of unknown function DUF538 family protein	Unknown
Os03g0131825			0.4	2.0	-2.5	Hypothetical protein	Unknown
Os02g0782550			1.4	7.6	-2.5	Hypothetical protein	Unknown
Os02g0138100	LOC_Os02g04550		0.4	2.0	-2.5	expressed protein	Unknown
