# Supplementary material for: Transcriptional response of rice flag leaves to restricted external phosphorus supply during grain filling in rice cv. IR64
Source: PLoS One. 2018 Sep 13;13(9):e0203654. doi: 10.1371/journal.pone.0203654 (PMC6136725; doi:10.1371/journal.pone.0203654)
Supplement: S5 Table — (PDF) [file pone.0203654.s008.pdf]

Supplementary Table S5. 32 PRGs analysed in the present study

No.	Gene ID	MSU ID	Name / Description	FPKM * (Jeong *et al.*[10]) 6 DAA	15 DAA	Log_2
1	Os01g0800500	LOC_Os01g58640	*OsPAP9b*	5.76	53.07	3.20
2	Os03g0848100	LOC_Os03g63074	*OsPAP15*	0.19	1.37	2.82
3	Os02g0678200	LOC_Os02g45520	*OsSPX-MFS2*	35.03	243.11	2.80
4	OS07G0187400	LOC_Os07g08970	Conserved hypothetical protein	44.01	288.27	2.71
5	Os04g0573000	LOC_Os04g48390	*OsSPX-MFS1*	6.11	35.69	2.55
6	OS03G0238600	LOC_Os03g13540	*OsPAP3c*, Ser/Thr protein phosphatase family protein, putative, expressed, Similar to Purple acid phosphatase	10.74	59.36	2.47
7	OS07G0165200	LOC_Os07g07080	regulator of chromosome condensation/beta-lactamase-inhibitor protein II, putative, expressed, Ankyrin domain containing protein.	8.59	35.30	2.04
8	OS02G0514500	LOC_Os02g31030	glycerophosphoryl diester phosphodiesterase family protein, putative, expressed, Similar to Glycerophosphoryl diester phosphodisterase (Fragment)	6.06	24.77	2.03
9	OS06G0291500	LOC_Os06g18820	serine threonine kinase, putative, expressed, Conserved hypothetical protein.	0.34	1.36	1.99
10	OS01G0142300	LOC_Os01g04920	*OsSQD2*, glycosyl transferase, group 1 domain containing protein, expressed	12.03	37.18	1.63
11	OS04G0185600	LOC_Os04g10690	*OsPT5*	0.30	0.90	1.60
12	OS01G0110100	LOC_Os01g02000	OsPHO1;1, PHO1;1	2.50	7.30	1.55
13	OS02G0802700	LOC_Os02g55910	monogalactosyldiacylglycerol synthase, putative, expressed, Similar to MGDG synthase type A	12.18	34.87	1.52
14	OS09G0554000	LOC_Os09g38100	*OsPT20*	1.80	5.00	1.47
15	OS03G0261800	LOC_Os03g15530	expressed protein, Protein of unknown function DUF3049 domain containing protein	12.71	33.58	1.40
16	OS01G0310100	LOC_Os01g20860	*OsPLDzeta2*, *OsPLDrho2*, phospholipase D. Active site motif family protein, expressed	3.35	8.74	1.38
17	OS01G0776600	LOC_Os01g56880	*OsPAP10a*, purple acid phosphatase precursor, putative, expressed	16.63	41.37	1.31
18	Os06g0643900	LOC_Os06g43640	*OsPAP26*	17.48	43.13	1.30

* FPKM values were retrieved from Jeong *et al.*[10].

Supplementary Table S5. Continued.

No.	Gene ID	MSU ID	Name / Description	FPKM * (Jeong *et al.*[10]) 6 DAA	15 DAA	Log_2
19	OS05G0557700	LOC_Os05g48390	*OsPHO2*	8.93	18.89	1.08
20	OS07G0134500	LOC_Os07g04210	Ser/Thr protein phosphatase family protein, putative, expressed, Similar to hydrolase/ protein serine/threonine phosphatase	9.82	20.74	1.08
21	OS08G0156600	LOC_Os08g06010	transporter, major facilitator family, putative, expressed, Major facilitator superfamily protein.	8.95	18.34	1.03
22	OS01G0557500	LOC_Os01g37690	*OsCAX1a, CAX1, OsCAX1*	19.79	38.98	0.98
23	OS03G0214400	LOC_Os03g11560	digalactosyldiacylglycerol synthase, chloroplast precursor, putative, expressed, Similar to Digalactosyldiacylglycerol synthase 2	5.74	11.04	0.94
24	OS09G0454600	LOC_Os09g28160	*OsPT19*	3.56	6.68	0.91
25	OS05G0358700	LOC_Os05g29050	*OsPLDrho1*	9.97	16.72	0.75
26	OS09G0528700	LOC_Os09g35940	cytochrome P450, putative, expressed, Similar to Cytochrome p450 (CYP78A9).	15.78	26.39	0.74
27	OS06G0140800	LOC_Os06g04880	serine threonine kinase, putative, expressed, Protein kinase, core domain containing protein	24.09	39.60	0.72
28	OS06G0603600	LOC_Os06g40120	*OsSPX1*	31.50	51.15	0.70
29	OS02G0202200	LOC_Os02g10780	*OsSPX2*	33.67	54.16	0.69
30	OS12G0576600	LOC_Os12g38750	*OsPAP1d*, Nucleotide pyrophosphatase/phosphodiesterase, putative, expressed	12.11	18.76	0.63
31	Os06g0129400	LOC_Os06g03860	*OsSPX-MFS3*	14.93	22.24	0.57
32	OS08G0433200	LOC_Os08g33640	Expressed protein, Conserved hypothetical protein.	34.26	50.60	0.56

* FPKM values were retrieved from Jeong *et al.*[10].
